# Low-Dose Near-Infrared Light-Activated Mitochondria-Targeting Photosensitizers for PDT Cancer Therapy

**DOI:** 10.3390/ijms23179525

**Published:** 2022-08-23

**Authors:** Wenyu Wu Klingler, Nadine Giger, Lukas Schneider, Vipin Babu, Christiane König, Patrick Spielmann, Roland H. Wenger, Stefano Ferrari, Bernhard Spingler

**Affiliations:** 1 Department of Chemistry, University of Zurich, Winterthurerstrasse 190, 8057 Zurich, Switzerland; 2 Laboratory for Advanced Fibers, Empa Swiss Federal Laboratories for Materials Science and Technology, Lerchenfeldstrasse 5, 9014 St. Gallen, Switzerland; 3 Institute of Molecular Cancer Research, University of Zurich, Winterthurerstrasse 190, 8057 Zurich, Switzerland; 4 Institute of Physiology, University of Zurich, Winterthurerstrasse 190, 8057 Zurich, Switzerland

**Keywords:** phthalocyanine, cancer treatment, photodynamic therapy (PDT), phototoxic index (p.i.), cisplatin, cytotoxicity, photocytotoxicity, crystal structure

## Abstract

Phthalocyanines (Pcs) are promising candidates for photodynamic therapy (PDT) due to their absorption in the phototherapeutic window. However, the highly aromatic Pc core leads to undesired aggregation and decreased reactive oxygen species (ROS) production. Therefore, short PEG chain functionalized A_3_B type asymmetric Pc photosensitizers (PSs) were designed in order to decrease aggregation and increase the aqueous solubility. Here we report the synthesis, characterization, optical properties, cellular localization, and cytotoxicity of three novel Pc-based agents (**LC31**, **MLC31**, and **DMLC31Pt**). The stepwise functionalization of the peripheral moieties has a strong effect on the distribution coefficient (log*P*), cellular uptake, and localization, as well as photocytotoxicity. Additional experiments have revealed that the presence of the malonic ester moiety in the reported agent series is indispensable in order to induce photocytotoxicity. The best-performing agent, **MLC31**, showed mitochondrial targeting and an impressive phototoxic index (p.i.) of 748 in the cisplatin-resistant A2780/CP70 cell line, after a low-dose irradiation of 6.95 J/cm^2^. This is the result of a high photocytotoxicity (IC_50_ = 157 nM) upon irradiation with near-infrared (NIR) light, and virtually no toxicity in the dark (IC_50_ = 117 μM). Photocytotoxicity was subsequently determined under hypoxic conditions. Additionally, a preliminarily pathway investigation of the mitochondrial membrane potential (MMP) disruption and induction of apoptosis by **MLC31** was carried out. Our results underline how agent design involving both hydrophilic and lipophilic peripheral groups may serve as an effective way to improve the PDT efficiency of highly aromatic PSs for NIR light-mediated cancer therapy.

## 1. Introduction

Photodynamic therapy (PDT) serves as a clinically approved non-invasive treatment for a broad range of cancers with selective and modulable cytotoxic activity [1,2,3]. PDT treatment requires the presence of three essential components (photosensitizer (PS), light, and oxygen). After application, a local irradiation of the tissue using light of an appropriate wavelength at the target tumor site activates the PS. Upon light irradiation, the PS can either transfer the energy from its excited state to molecular oxygen or directly react with biomolecules. Both pathways lead to the generation of cytotoxic reactive oxygen species (ROS) [4,5]. These locally generated ROS then react with the cellular microenvironment or organelles, leading to apoptosis, necrosis, or autophagy of the tumor cells. This can be accompanied by an acute local inflammatory reaction that leads to the removal of dead cells, restoration of normal tissue homeostasis and occasionally systemic immunity [2,6]. Since PSs are ideally only activated upon intentional irradiation with light, they induce minimal toxic side effects and the PDT treatment can be repeated without the appearance of an accumulative toxicity. Therefore, PDT has been an attractive alternative treatment option for different severe cancers, while improving the survival rate without compromising the life quality of the affected patients [2,7].

Despite the many merits, PDT has not yet been widely accepted in clinics due to certain limitations of the reported PSs. Phthalocyanines (Pcs) are considered promising candidates for PDT because of their intense optical absorption in the red region of the visible light spectrum [8,9,10,11,12,13]. Their absorption spectra perfectly match the desired PDT phototherapeutic window of 650–850 nm [14,15,16], which has an excellent penetration depth in human tissue [17]. Normally, most of the PSs have an extended aromatic ring system, which makes them highly hydrophobic and susceptible to π–π stacking. This then results in poor solubility in aqueous environments, a rapid clearance from the blood circulation, and therefore a low bioavailability. Additionally, the high aromaticity results in undesired aggregation, leading to a quenching of the singlet oxygen generation and thereby to a reduced therapeutic effect [18]. An important parameter to evaluate the effect of PSs is the phototoxic index (p.i.), i.e., the ratio of dark to light toxicity [19,20,21,22,23,24,25,26,27]. Reducing the lipophilicity and minimizing aggregation of the planar PS, for example by PEGylation, plays a critical role for improving PDT therapeutic efficiency and improving the p.i. [28,29,30,31,32,33,34,35,36,37,38,39]. Hence, exploring suitable designs to modify PSs for improving their amphipathicity is of high importance.

Herein, we have designed amphiphilic peripheral units to reduce the aggregation and self-quenching of the A_3_B asymmetric type Pc PSs. Through stepwise modification, three PSs were obtained, each with very different distribution coefficients (log*P*) and, therefore, differential cellular uptake behaviors. In this work, we report novel, amphipathic Pc PSs **LC31**, **MLC31**, and **DMLC31Pt** and their photocytotoxicities under normoxic and hypoxic conditions.

## 2. Results

**Figure 1:**
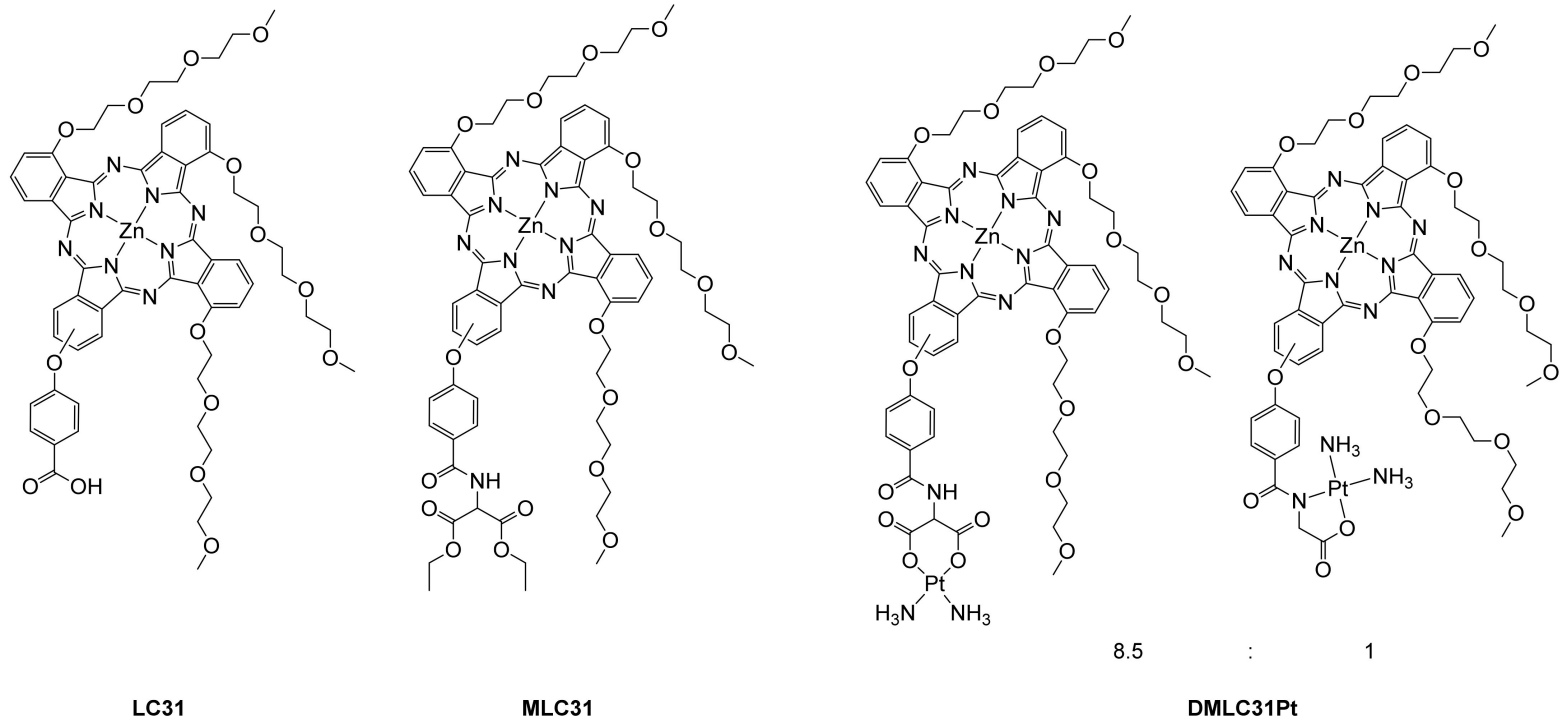
Studied structures: **LC31**, **MLC31**, and **DMLC31Pt**.

**Scheme 1:**
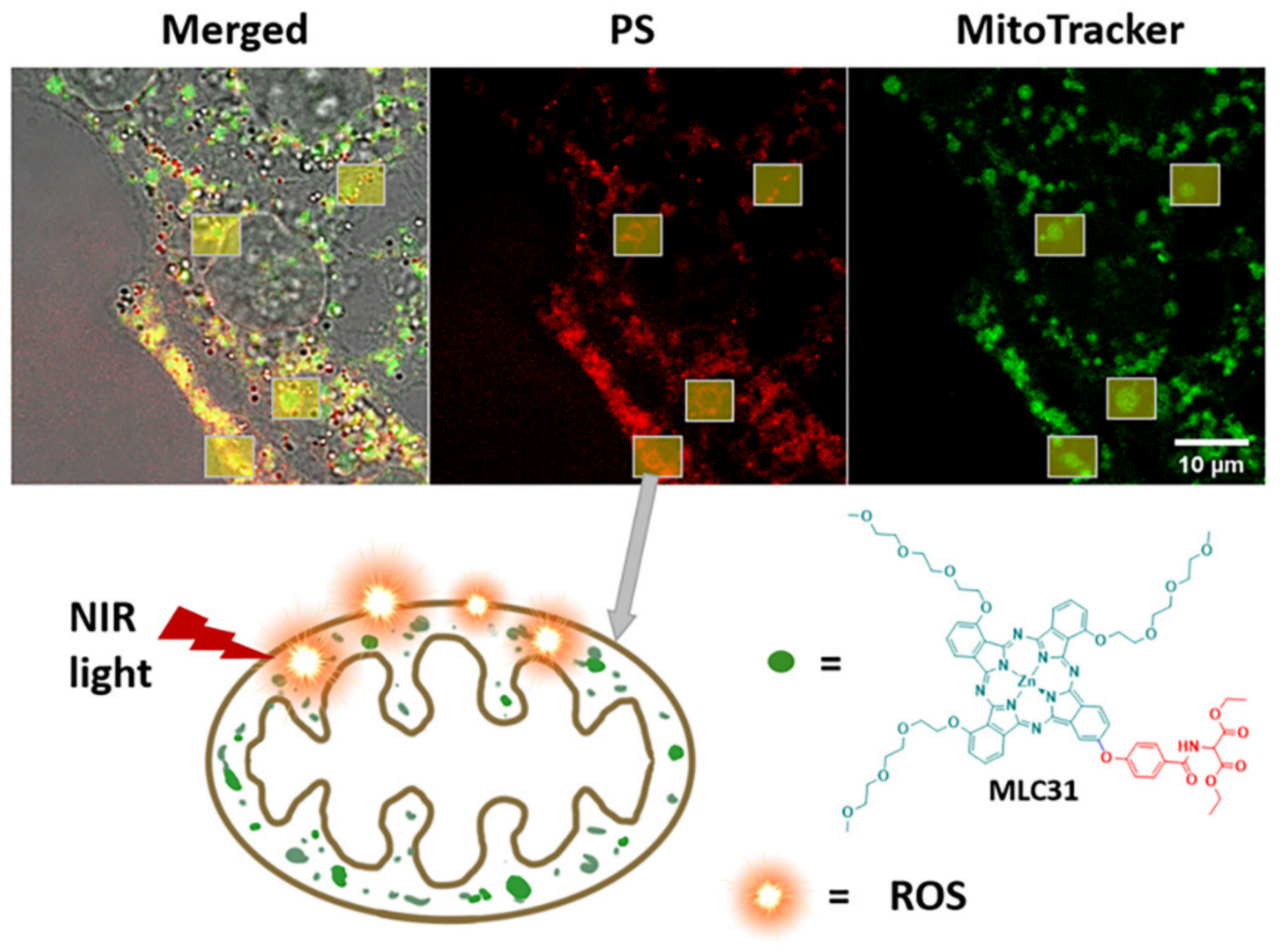
The light-activated function of PS **MLC31**. Top panel: **MLC31** uptake into mitochondria of HeLa cells (200 nM, 4 h incubation). Bottom panel: the proposed cellular response of **MLC31** (marked in green) within a mitochondrion of a cancer cell after activation by NIR light (**left**), and chemical structure of photosensitizer **MLC31** (**right**).

A series of three novel A_3_B type Pcs with three glyoxylic substitution groups and one functional moiety was synthesized (Figure 1 and Scheme S1, Supporting Information). The phthalonitriles 3 and 6 (in a ratio of 3:1) were reacted with zinc(II) acetate and 1, 8-diazabicyclo [5.4.0]undec-7-ene (DBU) as a base in a macrocyclization reaction in *n*-pentanol to give the novel A_3_B disubstituted non-symmetrical zinc(II) Pc **LC31** (Scheme S1) according to reported procedures [9,40]. **MLC31** was obtained through a coupling of **LC31** with amino diethylmalonate, which has excellent and specific mitochondria-targeting properties in cancer cells, leading to an efficient NIR light-induced cytotoxicity (Scheme 1). After deprotection of **MLC31**, the acid moiety was coordinated further with activated cisplatin in order to obtain **DMLC31Pt**. Analysis by ^195^Pt-NMR showed that the obtained platinated PS **DMLC31Pt** is a mixture of N,O—Pt and O,O′—Pt chelates in a ratio of about 8.5:1 (Figure S2 and Table S1, Supporting Information). In the N,O—Pt chelate (−2102.0 ppm) the platinum is coordinated to the nitrogen of the amide group and to the carboxylate oxygen atom of the decomposed malonate moiety (**DMLC31Pt**), while in the O,O′—Pt chelate (−1760.4 ppm) the platinum is coordinated to the two carboxylate oxygen atoms of the malonate moiety. The photophysical properties, ability to generate ROS, photocytotoxicity, and cellular uptake properties of these PSs, in particular **MLC31**, have been studied and compared in order to evaluate the different modifications.

**Figure 2:**
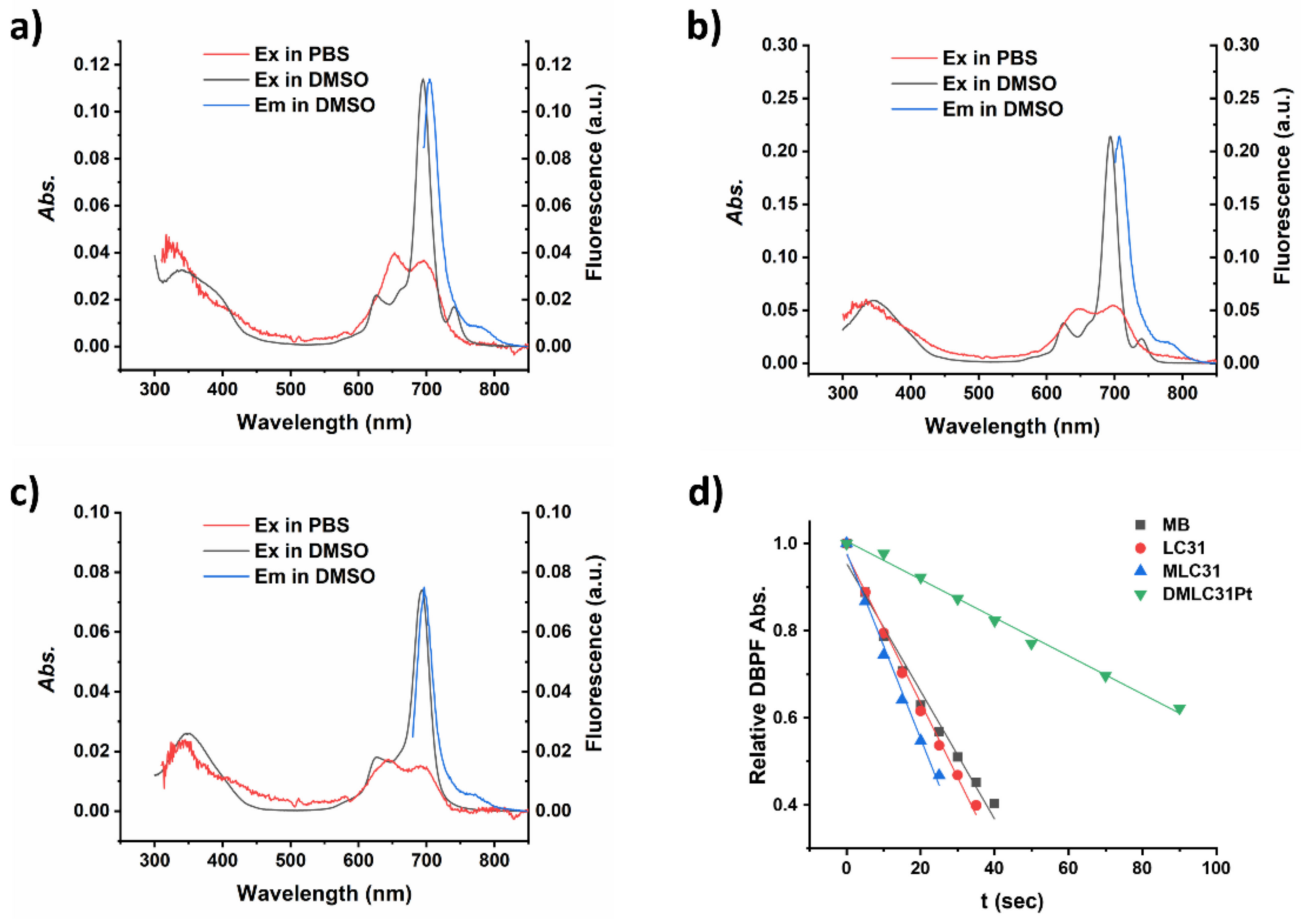
UV-Vis absorption spectra of (**a**) **LC31**, (**b**) **MLC31**, and (**c**) **DMLC31Pt** in DMSO or PBS, and the corresponding normalized emissions in DMSO. Emission in PBS is not shown because none could be observed. (**d**) Plots of optical density change (ΔOD) of DPBF in methanol monitored at 410 nm versus the irradiation time. Light of wavelengths in the range of 600–720 nm with a power intensity of 20 mW cm^−2^ was applied with a halogen light source.

Pcs **LC31**, **MLC31,** and **DMLC31Pt** all display a B-band at approximately 340 nm and a Q-band at approximately 695 nm. Of all the tested PSs, **MLC31** has the highest Q-band extinction coefficient (*ϵ*) in all tested solvent systems (Table 1), especially in aqueous environments such as phosphate-buffered saline PBS (57,900 M^−1^ cm^−1^, compared to 37,400 M^−1^ cm^−1^ in the case of **LC31** and 19,500 M^−1^ cm^−1^ in the case of **DMLC31Pt**). Yet for all PSs, an aggregation tendency in PBS was observed, leading to split, broadened and blueshifted Q-bands when compared to solvent systems like dimethyl sulfoxide (DMSO) or MeOH (Figure 2a–c and Figure S3). In terms of fluorescence, **MLC31** displayed emission in DMSO and MeOH but not in PBS. In DMSO, the peak is at 704 nm, which corresponds to a 7 nm *Stokes* shift. The property of strong fluorescence emission in the NIR range allows for the tracking of thesePSs in cell localization studies via fluorescence microscopy and in vivo during the treatment.

ROS, especially singlet oxygen (^1^O_2_), are considered to be the main cytotoxic agents in PDT, therefore the ^1^O_2_ quantum yields (Φ_∆_) of the PSs have been investigated by comparison with a standard [41,42]. The Φ_∆_ upon irradiation with light was evaluated with a steady-state method while monitoring the PS-mediated photo-oxidation of 1,3-diphenylisobenzofuran (DPBF) using methylene blue (MB) as a standard (Φ_∆_ of 0.52 in MeOH [43]). Therefore, a solution of the corresponding Pc-based PS containing DPBF was irradiated using a halogen lamp with a 600 nm long-pass filter over a time period of 0 to 150 s while the decrease of the absorption band of DPBF at 410 nm was monitored. The Φ_∆_ was then calculated by comparing the decrease to that of MB (Figure S4, Supporting Information) through plotting the optical density change (ΔOD) of DPBF against the irradiation time (Figure 2d). As illustrated in Figure S4, the absorbance of the mixtures of the corresponding PS and DPBF decreased quickly at 410 nm upon irradiation with light, indicating an efficient generation of ^1^O_2_. In the control experiment without PSs, no significant decrease could be observed at 410 nm during an irradiation time of 1 min. The Φ_∆_s for **LC31**, **MLC31**, and **DMLC31Pt** in methanol were determined to be 0.49, 0.84, and 0.39, respectively. Surprisingly, the Φ_∆_ of **MLC31** is clearly higher compared to that of **LC31** and **DMLC31Pt**. Possible reasons for this observation are the higher *ϵ* of **MLC31** at 700 nm and the slightly different aggregation form, described below. Additionally, the ability of all three Pc-based PSs to generate hydroxyl radicals (^•^OH) upon irradiation with NIR light was determined with help of the APF sensor (Supporting Information). Interestingly, **LC31** generated the most ^•^OH, followed by **DMLC31Pt** and **MLC31** (Figure S5).

The aggregation of highly aromatic dye molecules plays an important role in energy and electron transfer and light harvesting systems [45]. It is well known that Pcs tend to form aggregates caused by the strong π–π interactions between the planar macrocycles in solution. The relative geometry of the macrocycles reflects on the spectroscopic behavior of the aggregates. Previous reports have shown that the Q-band of aggregates is split compared to well-dispersed single molecules (Figure 2) when the angle between the polarization axes of monomers differs or the monomer center of the aggregate shifts. If the Pc centers are overlapping, with the ring systems being stacked in a parallel way, the aggregates are called H-type aggregates, which lead to a blueshifted band. On the other hand, when the molecules are aggregated in a head-to-tail form and the centers are further apart, the aggregates are called J-type aggregates and the band is redshifted [46]. The co-facial arrangement, which is common in most Pc aggregates, generally yields a blueshifted H-aggregates, whereas an edge-to-edge arrangement of the J-aggregates is rare. Compound **MLC31** containing triethylene glycol substituents resulted in an unusually mixed Q-band with a redshifted peak (Q^−^ = 698 nm, compared to Q = 694 nm) and a blueshifted peak (Q^+^ = 646 nm) upon aggregation in aqueous media (Figure 2b). This suggests a co-existence of H-type and J-type aggregates. In contrast to **MLC31**, **LC31** showed no redshift (Q^−^ = 696 nm, compared to Q = 696 nm) but only a blueshifted peak (Q^+^ = 653 nm) upon aggregation in aqueous media (Figure 2a), suggesting the existence of the H-type aggregates only. A similar behavior was displayed by **DMLC31Pt**, where Q^−^ = 693 nm (compared to Q = 693 nm) was observed, and a blueshifted peak at 644 nm.

In sharp contrast to the protonation of the porphyrin rings at the pyrrole nitrogen atoms, Pcs can undergo protonation at two different sites on the isoindole nitrogen atoms, as well as the *meso*-nitrogen atoms [47]. To elucidate the protonation site of **LC31** in solution, the absorption spectral changes upon addition of TFA to the solution of **LC31** in CHCl_3_ were measured. The titration of **LC31** with TFA in CHCl_3_ allowed observation of the one-step spectral change, with one isosbestic point at 705 nm in the case of **LC31** (Figure S6a). The split and shifted Q-bands indicate spectral changes accompanying the mono-protonation of **LC31**. These spectral changes could be attributed to the mono-protonation of the azomethine nitrogen bridges in the ZnPc ring, as according to previous reports [48,49], resulting in progressive redshifts and loss of symmetry. This apparent one-step mono-protonation is ascribed to the high basicity of the Pc *meso*-nitrogen bridge in the structural backbone, similar to mono-protonated porphyrins [50]. The equilibrium constant *K* of the Pc backbone mono-protonation was determined to be 7.1 ± 0.1 × 10^4^ M^−1^ for **LC31** in CHCl_3_ (Figure S6b) according to Equation (1):

(1) $$\mathrm{LC}31+\mathrm{HA}\;\overset{K}{\Leftrightarrow }\;[\mathrm{LC}31H^{+}A^{-}]$$

The redshift is a result of the reduction of the HOMO–LUMO gap in the electronic structure. The a_1u_ orbital has a nodal plane through the *meso*-nitrogen atoms [49,51]. The LUMO, unlike the HOMO, has a significant electron density on the azomethine nitrogen atoms. Engaging the nitrogen lone pairs withdraws electrons from the LUMO, thereby stabilizing it relative to the HOMO, whose energy is conserved. While mono-protonation of ZnPc was successful, TFA did not lead to the di-protonation of the ZnPc, compared to similar reports. For a di- or even tri-protonation, sulfuric acid might be used.

### 2.1. Photocytotoxicity

A successful PS for PDT exhibits an efficient off–on cytotoxicity in the absence and presence of the light used for the activation of the PS. Therefore, the cytotoxicity of the Pc-based compounds was evaluated against different cell lines both under irradiation and in the dark. Compounds **LC31**, **MLC31**, and **DMLC31Pt,** as well as cisplatin were screened against several cancer cell lines representative of human cervical cancer (HeLa), cisplatin-sensitive human ovarian carcinoma (A2780), cisplatin-resistant ovarian endometrioid adenocarcinoma (A2780/CP70), and non-cancerous fibroblast (MRC-5) cell lines. The cytotoxicity parameters are reported in **Table 2** in terms of the determined IC_50_ value (the median half-maximal growth inhibitory concentration calculated from dose–survival curves (Figure S7), obtained after 4 h incubation with respective compounds, 20 min of irradiation with a white light projector (filter cut-off below 600 nm, light intensity 5.8 mW cm^−2^, light dose: 6.96 J cm^−2^) and further 72 h of incubation). Overall, the tested agents were found to be much more active towards the cancerous cell lines after light activation compared to cisplatin and to the condition without activation by light.

**MLC31** was obtained by coupling **LC31** with amino-diethyl-malonic ester, thereby not only improving the lipophilicity, but also attaching a functional group. Surprisingly, **MLC31** showed the lowest IC_50_ value of the three tested Pc-based agents in HeLa cells after irradiation with NIR light (9 nM, Table 2), in comparison to 5.2 μM in the dark. The p.i. of **MLC31** is around 578. The IC_50_ value for **LC31** is 426 nM under light irradiation, lower than the IC_50_ value of cisplatin (1.7 μM). The p.i. of **LC31,** however, is over 30. In this context, it is interesting to note that Xue et al. found that their mono-PEGylated Pcs were much more toxic against HepG2 cells (IC_50_ values in the range of 12–17 nM) than the analogue Pcs, which were four-fold substituted with the same PEG substituent (IC_50_ values between 8 and >40 μM) [32]. After a coupling with cisplatin, **DMLC31Pt** might offer a double-functional chemotherapy/PDT mode of action. Yet, the observed cytotoxicity did not give solid evidence of an improved therapeutic efficiency of such a design. The IC_50_ values of **DMLC31Pt** upon light irradiation and in the dark tested in the HeLa cell line are 775 nM and 8.0 μM, respectively, with the p.i. being around 10. In conclusion, the observed phototoxicities of the three studied PSs could be positively correlated with their Φ_∆_s, but not with their abilities to generate ^•^OH. This indicates that ^1^O_2_ is the dominant agent responsible for the phototoxicity of **LC31**, **MLC31**, and **DMLC31Pt**.

### 2.2. DNA Damage

To gain mechanistic insights into the cellular effect of **DMLC31Pt**, we examined the phosphorylation of Ser139 of histone variant H2AX to give γH2AX, an established marker of the DNA damage response (DDR) [52]. An immunofluorescence-based method was used to detect γH2AX according to an established protocol [53]. HeLa cells incubated with **DMLC31Pt** for 16 h but not activated by NIR light showed only minor formation of γH2AX, while cells treated with **DMLC31Pt** and activated by NIR light showed a more intense γH2AX signal (Figure S8). Considering that **DMLC31Pt** is predominantly localized in the cytoplasm, generation of γH2AX in response to light irradiation of **DMLC31Pt** could be either triggered by low amounts of compound entering the nuclei or be a consequence of the activation of cell death pathways by irradiated **DMLC31Pt** that is present outside the nucleus.

A comparison of the relative cytotoxicities of **LC31**, **MLC31**, **DMLC31Pt**, and cisplatin was also made between the A2780 cell line and cisplatin-resistant A2780/CP70 cell lines (Figure S7). As expected, cisplatin resulted in no obvious cytotoxicity difference upon irradiation with light. Notably, after light irradiation at a very low intensity (2.0 W cm^−2^) for 20 min, the tested agents were found to be much more active in the cisplatin-resistant cell line (**MLC31** with IC_50_ around 157 nM) than cisplatin itself (129 μM), which indicates its therapeutic potential, particularly in light of the worrying emergence of cisplatin resistance in tumors [54]. Moreover, the finding that **MLC31** was found to be less cytotoxic than cisplatin under dark conditions in the non-cancerous MRC-5 cell line also implies that **MLC31** has a better therapeutic profile when compared to cisplatin.

The IC_50_ phototoxicity values after activation by NIR light irradiation follow the general trend of **LC31** ≈ **DMLC31Pt** >> **MLC31** (Table 2). It is surprising to note that **MLC31**, which is structurally very similar to **LC31** and **DMLC31Pt**, displayed quite different IC_50_ values than the latter two PSs (>5-fold for the A2780/CP70 cell line, >10-fold for the A2780, and >80-fold for the HeLa cell line) upon light irradiation. Interestingly, the photo-induced cytotoxicity of these PSs correlates rather well with the production of ^1^O_2_ (Table 1). In order to evaluate the action of the best PS, **MLC31**, against HeLa cells further, a comparison between its phototoxicity and those of the best molecular Pc-based PSs reported in literature was made (Table 3) [55]. This shows a clear trend that an increasing positive charge leads to a more phototoxic PS. However, the neutrally charged **MLC31** is clearly the most toxic Pc in the comparison series.

### 2.3. Cellular Uptake of LC31, MLC31, and DMLC31Pt

**Figure 3:**
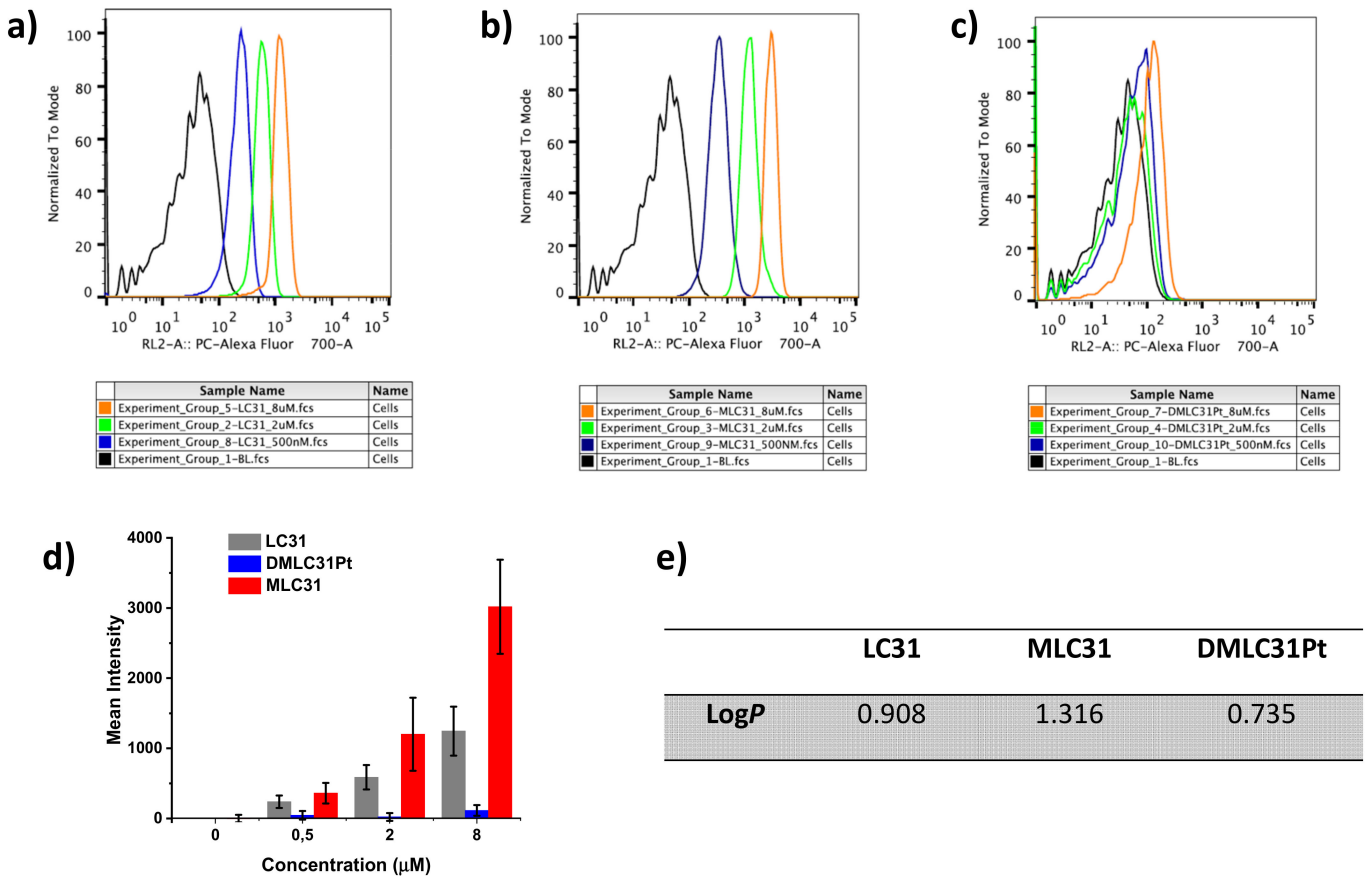
Flow cytometric histogram plots of the cellular uptake of (**a**) **LC31**, (**b**) **MLC31**, and (**c**) **DMLC31Pt**. HeLa cells were incubated with PSs (0 nM, 500 nM, 2 μM, and 8 μM) for 4 h. (**d**) Quantitative analysis of the amount of PS taken up by flow cytometry. (**e**) Distribution coefficients between *n*-octanol and PBS obtained using the *shake-flask* method.

HeLa cells were seeded in 6 cm dishes and three different concentrations (500 nM, 2 μM and 8 μM) of **LC31**, **MLC31**, and **DMLC31Pt** were added in triplicates, respectively. After 4 h of incubation with the PSs, the cellular uptake was investigated by flow cytometry. As shown in Figure 3, an intracellular accumulation of PSs was clearly observed, and the fluorescence intensity exhibited an increasing tendency with the increase of the PS concentrations (Figure 3a–c), consistent with the quantitative analysis of the mean fluorescence intensity (Figure 3d). At all of these tested concentrations, **MLC31** showed the highest cellular fluorescence intensity, which indicates the highest cellular uptake of these three PSs. The cellular uptake of the PSs was evaluated at two different temperatures (4 °C and 37 °C) to determine whether the internationalization occurred in an energy-dependent manner [64]. Compared to 37 °C, the cellular uptake of the PSs was significantly inhibited at 4 °C, according to the fluorescence intensity analyzed by flow cytometry (Figure S9). This indicates that **MLC31** was internalized into the cells via an energy-dependent active pathway.

### 2.4. Distribution Coefficient (logP)

The lipophilicity of a compound has a strong influence on its cellular uptake, localization, and further biodistribution. Chemical drugs have to cross a series of barriers in the body by either passive diffusion or a carrier-mediated transport [65]. The distribution coefficient (log*P*), the logarithm of its partition coefficient between *n*-octanol and water (log(c_octanol_/c_water_)), is used as one of the principal parameters to evaluate the lipophilicity of chemical compounds, therefore influencing the pharmacokinetic properties of drugs. Low hydrophilicities and thus high log*P* values normally indicate poor absorption or permeation. The lipophilicity of the Pc agents reported here was determined by measuring the distribution coefficients in PBS at a pH = 7.4, an approximation of physiological conditions, using the *shake-flask* method [66,67]. As shown in Figure 3e, **MLC31** has the highest lipophilicity of the series due to the presence of the ethyl malonic ester moiety, with a log*P* value around 1.316. **LC31** has a log*P* value of 0.908, and **DMLC31Pt** has the lowest log*P* value of 0.735. These distribution coefficient values are in line with the cellular uptake measured using flow cytometry, as a certain lipophilicity of **MLC31** leads to higher cellular uptake. These results also correlate with the NIR light-induced intracellular ROS intensities, showing that **ML31** has the highest ability to generate ROS in cells (Figure S10).

### 2.5. Cell Localization via In Vitro Fluorescence Evaluation

**Figure 4:**
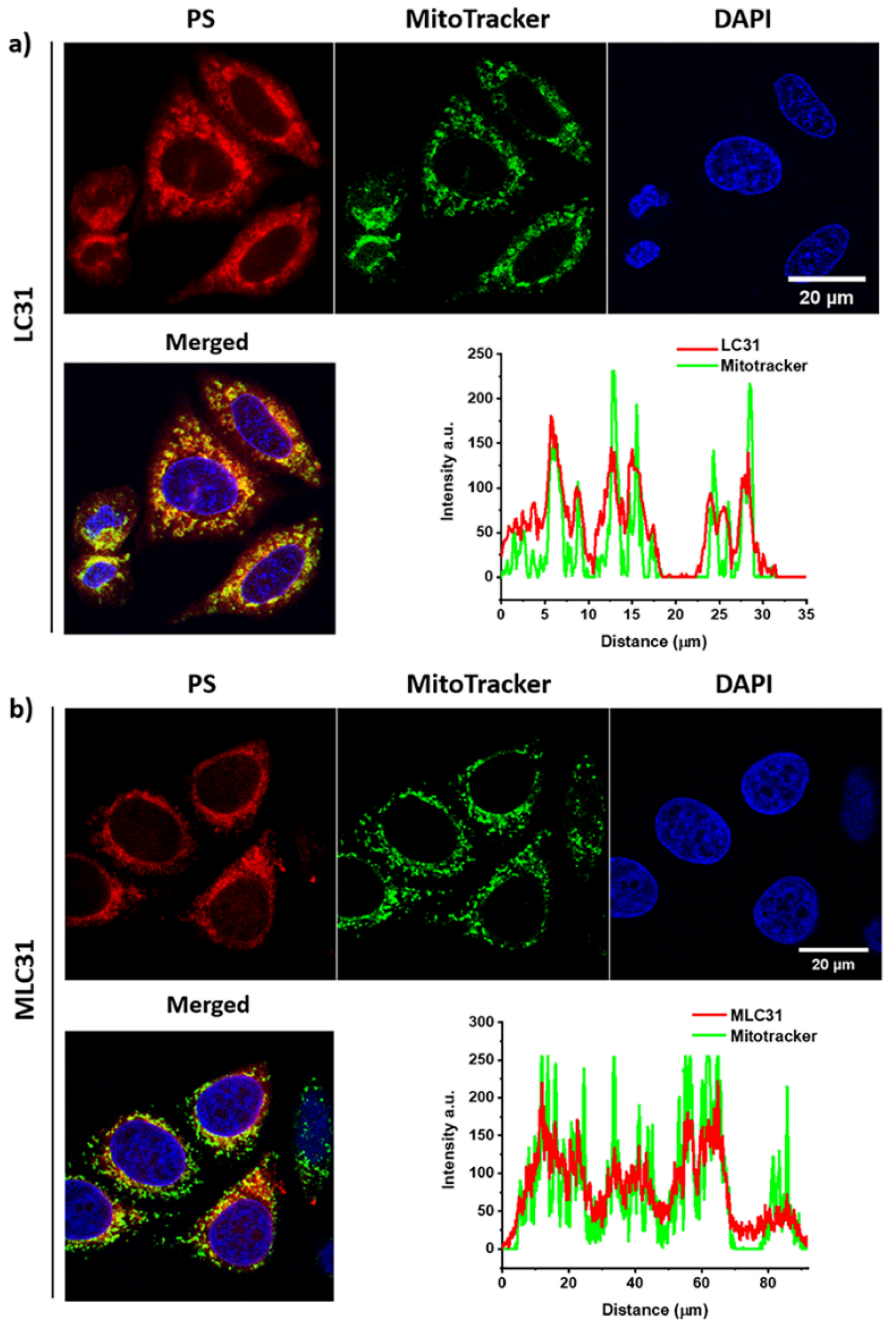
Subcellular localization of (**a**) **LC31** and (**b**) **MLC31** by MitoTracker Deep Red FM and DAPI staining. HeLa cells were incubated with 1 μM **LC31** or 500 nM **MLC31** for 2 h. The PSs were visualized upon excitation at 633 nm. Images merging and multi-channel intensity line profile plots were obtained using the software Fiji [72].

The mechanism of PDT-induced cell death via apoptosis or necrosis is highly dependent upon the localization of the PS within the cell and the amount of cytotoxic ^1^O_2_ generated [68]. Some evidence suggests that a PS, which is localized in the mitochondria or the endoplasmic reticulum (ER), is a better inducer of apoptosis, whereas PS localized in the plasma membrane or lysosomes is more conducive to necrosis [69]. The diverse photophysical properties of metal–Pc complexes, especially the fluorescent properties, can be utilized to study their cellular accumulation, biodistribution, and metabolic pathways. This, in turn, allows for decision-making on whether they are ideal model compounds to construct novel theragnostic platforms combining diagnostic and therapeutic purposes. As a first step towards elucidating the cellular uptake action of **MLC31**, favorable NIR emission has been used to evaluate the localization of the Pc-based agents in HeLa cells. Cellular localization of **LC31**, **MLC31**, and **DMLC31Pt** was assessed by fluorescence confocal microscopy. All investigated PSs were effectively taken up by HeLa cells, and the emerging fluorescence emission showed that **LC31** and **MLC31** co-localized well with the MitoTracker dye (Figure 4 and Figure S11). This shows that **LC31** and **MLC31** tend to specifically accumulate in mitochondria, more precisely in the mitochondrial outer membrane, while **DMLC31Pt** accumulated in the cytoplasm and nucleoli (Figure S8). Unlike **DMLC31Pt**, **MLC31** did not accumulate in the cell nucleus, therefore limiting potential DNA damage that could be carcinogenic or lead to the development of resistant clones. Within PDT, the primary pathway for the execution of mitochondria-targeting PS-induced cell death has been attributed to apoptosis [69,70]. Induction of apoptosis by **MLC31** was observed by detecting changes of the cell morphology from image acquisitions in HeLa cells. As shown in Figure S12, a longer light irradiation (633 nm) of cells treated with **MLC31** resulted in increasing abnormal cell morphology within few minutes. More specifically, plasma membrane blebbing (zeiosis) occurred, one of the defining characteristics of apoptosis [71]. The fluorescent signal of **MLC31** remained in the mitochondria during the procedure of the image acquisition.

### 2.6. Detection of the Loss of the Mitochondrial Membrane Permeabilization

Mitochondria play a central role in cellular homeostasis, not only providing energy from ATP by the process of oxidative phosphorylation, but also by decisively regulating the intrinsic pathway of apoptosis [73]. The integrity of the mitochondrial membrane, and especially an intact electrochemical gradient, are responsible for ATP production, while dysfunction of the latter is linked to the release of pro-apoptotic factors [74,75]. The hallmark of mitochondrial dysfunction is the loss of the mitochondrial membrane potential (MMP, Δ*Ψ*_m_). The collapse of the Δ*Ψ*_m_ coincides with the opening of the mitochondrial permeability transition pore, leading to the release of soluble proteins such as cytochrome c (cyt c) into the cytosol. The study of the Δ*Ψ*_m_ is essential for an integrated appraisal of mitochondrial function, since it reflects differences in the electrical potential and represents the main component of the proton electrochemical gradient—accounting for more than 90% of the total available respiratory energy. The collapse of Δ*Ψ*_m_ in tumor cells constitutes one of the goals of anticancer chemotherapy. As the aforementioned results confirmed that **MLC31** localizes mainly in mitochondria, an attempt to better understand the kinetics and consequences of apoptotic damage was performed though the detection of the cellular MMP with or without irradiation with NIR light.

**Figure 5:**
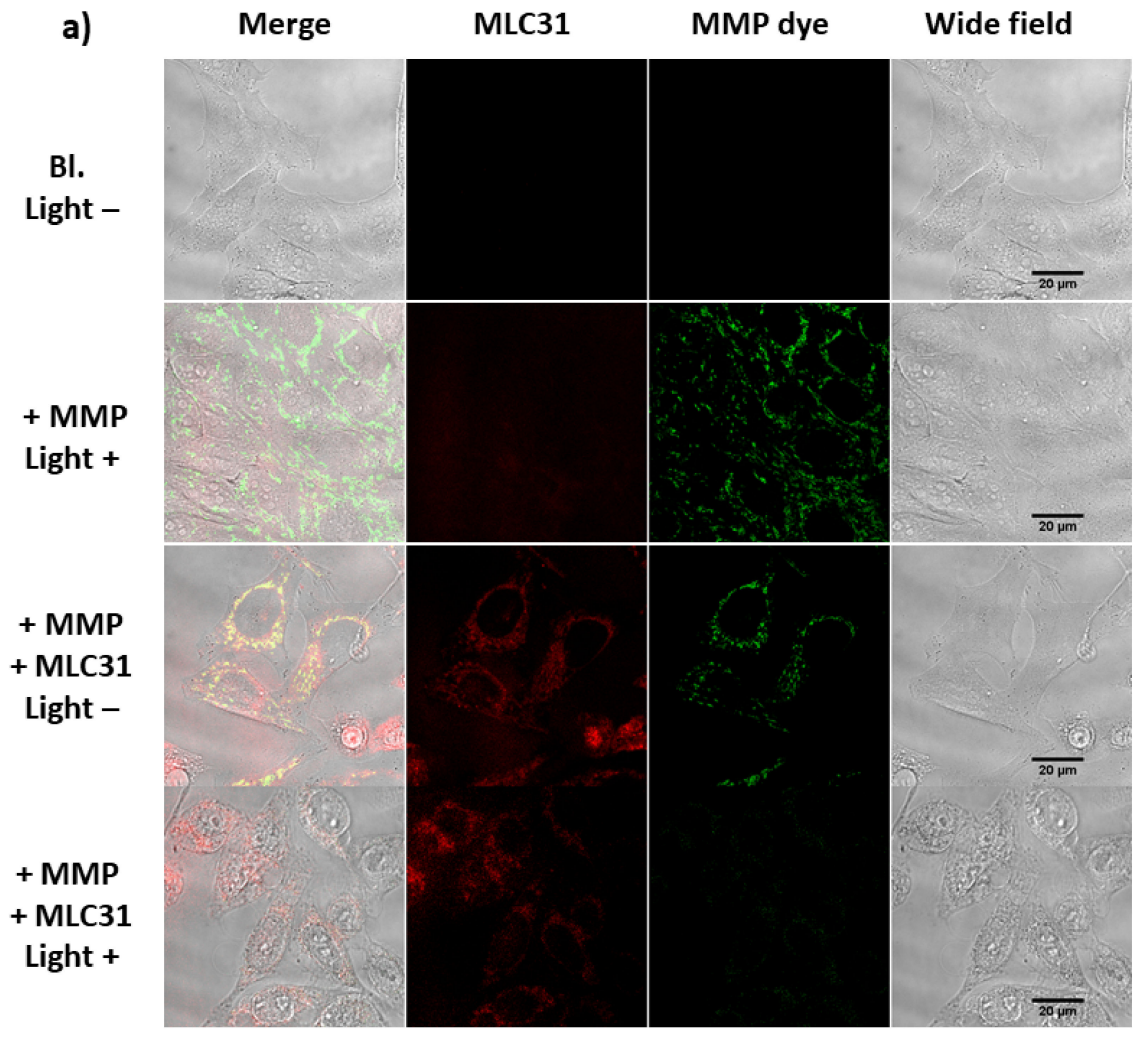

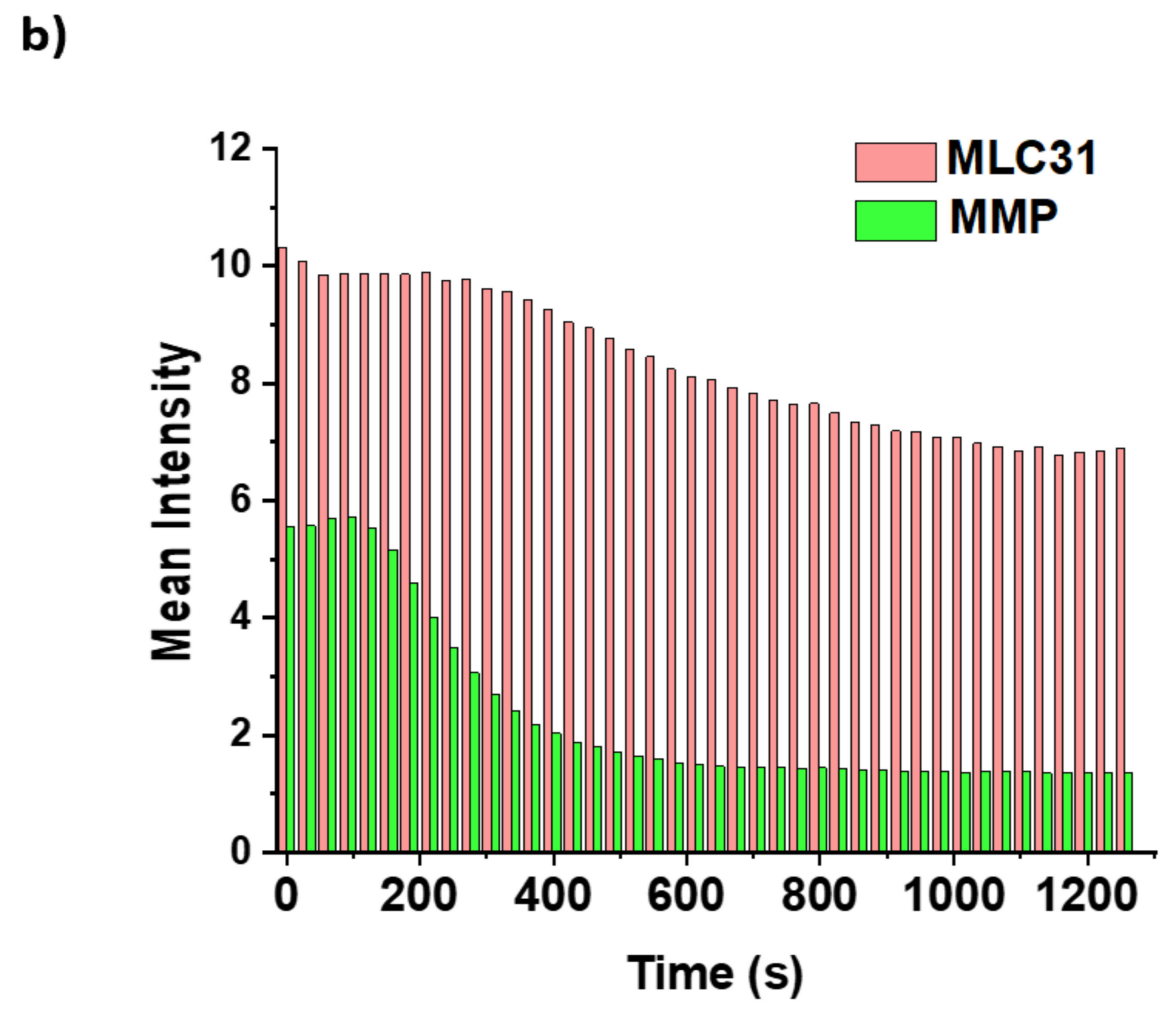
(**a**) NIR light-induced Δ*Ψ*_m_ change measured by the MMP assay. (**b**) Timelapse of intensity variation analysis of **MLC31** and Δ*Ψ*_m_ channels obtained through the ROI Manager for intensity analysis using Fiji [72].

As shown in Figure 5, no detectable loss in the Δ*Ψ*_m_ was found without irradiation by light for cells treated with **MLC31**, or in the control condition without administration of the compound and activation by NIR light. However, **MLC31** in the presence of NIR light led to an extensive decrease in the Δ*Ψ*_m_ together with a decrease in the fluorescence signal, which occurred within few minutes (Figure 5b, Figure S12 and Figure S13). The Δ*Ψ*_m_ collapse leads to release of cyt c into the cytosol, starting the apoptotic pathway.

### 2.7. Effects under Hypoxia

Solid tumors are known to contain regions with oxygen deficiency (hypoxia), which triggers reprogramming of tumor metabolism and angiogenesis, leading to metastasis and decreased patient survival [76]. Tumor hypoxia not only increases the resistance to radiotherapy and chemotherapy, but also plays a critical role in PDT, because the ROS generation is oxygen dependent. In order to evaluate the effect of oxygen availability, the light irradiation-induced activity of **MLC31** in HeLa cells was investigated under normoxic (18.6% O_2_) and hypoxic (0.2% O_2_) conditions.

**Figure 6:**
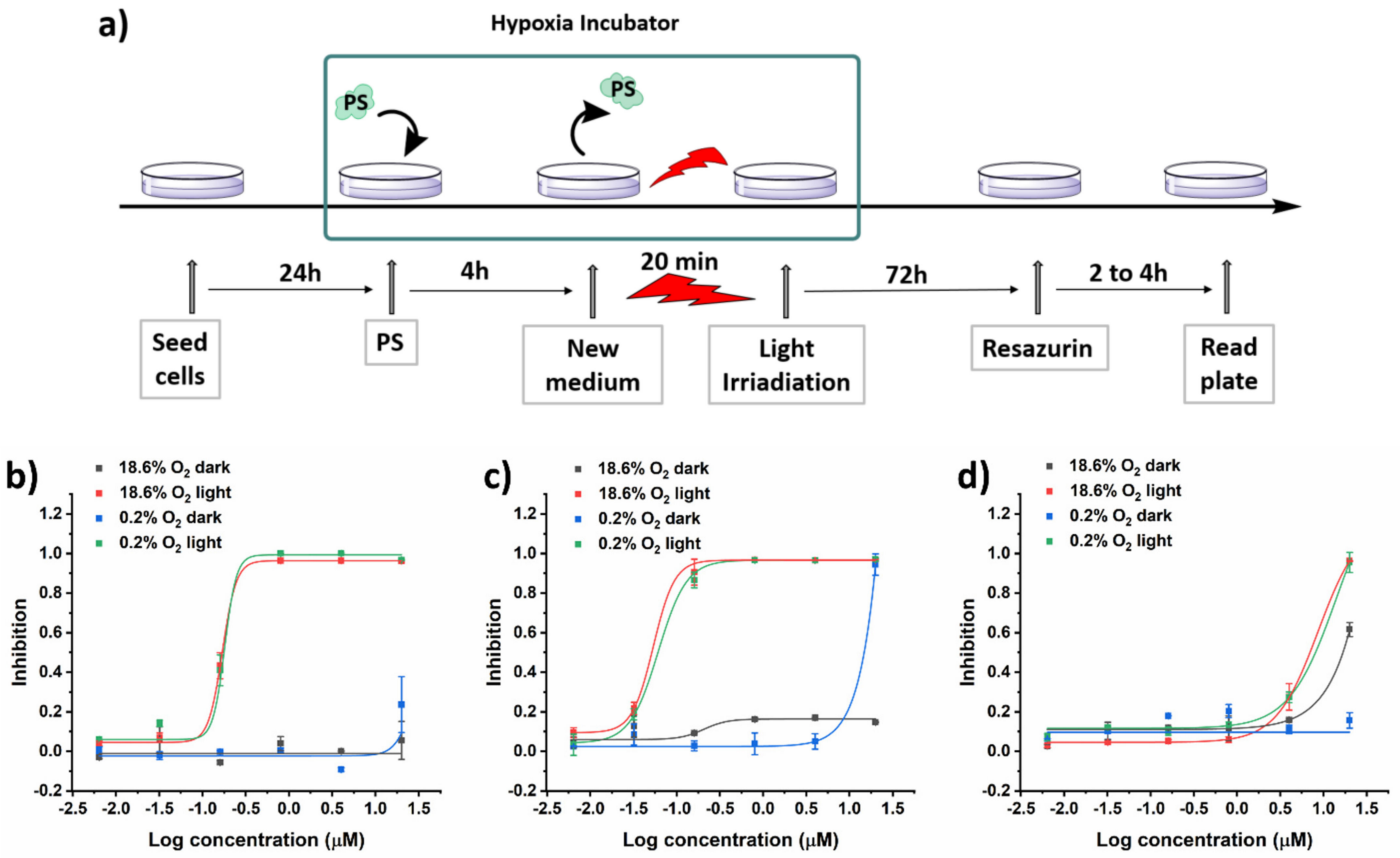
(**a**) Schematic protocol of the cytotoxicity determination in HeLa cells under hypoxic conditions. Cells were treated with (**b**) **LC31**, (**c**) **MLC31**, and (**d**) **DMLC31Pt**, respectively, for 4 h under hypoxic or normoxic conditions (inhibition of viability shown, LED light source of 660 nm, light intensity = 9 mW cm^−2^, light dose = 10.8 J cm^−2^).

As shown in Figure 6a, HeLa cells were incubated with PS and subjected to photoirradiation under hypoxic conditions before being returned to normoxia. While for all tested compounds the inhibition efficiency decreased only slightly under hypoxic conditions (Figure 6b–d and Table 4), a high level of cytotoxicity was still observed, even at an oxygen concentration as low as 0.2%, with IC_50_ values of 56 nM and a p.i. of 74 (Figure 6). The different IC_50_ values obtained in this series of experiments compared with the ones reported in Table 1 can be explained by the use of different light sources (a projector with 600 nm cutoff long-pass filter was used to obtain the results reported in Table 1, while a 660 nm LED light source was used for the results reported in Table 4). These are promising results since tumor hypoxic microenvironments exhibit a wide range of different oxygen partial pressures, corresponding to gas phase concentrations in the range of 0 to 5%, while **MLC31** is effective even at an oxygen concentration of 0.2% [77]. This suggests that **MLC31** is able to ablate cells even in harsh hypoxic environments. Based on these results, we suggest that **MLC31** could potentially also be highly effective in solid tumors.

## 3. Materials and Methods

### 3.1. General Materials and Instrumentation

Unless otherwise stated, all chemicals were of reagent grade and purchased from Sigma-Aldrich (Darmstadt, Germany), Merck (Darmstadt, Germany), Alfa Aesar (Kandel, Germany), or Fluorochem (Hadfield, United Kingdom). Reactions were carried out under N_2_ and monitored for completion by analysing a small sample by TLC or UPLC. Solvents for reactions were of p.a. grade. Evaporation of the solvents in vacuo was done with a rotary evaporator at the given bath temperature and pressure. A vacuum line and *Schlenk* glassware were employed when reactions had to be carried out under a dry atmosphere. Assemblies were protected from light, if necessary, by wrapping them with aluminium foil. Antibodies: rabbit polyclonal antibodies to phosphorylated histone H2AX (S_139_) from Cell Signalling Technology (Beverly, MA, USA) were used. HRP-conjugated anti-mouse and anti-rabbit secondary antibodies were obtained from Amersham-Biosciences/GE-Healthcare (Otelfingen, Switzerland). Alexa Fluor^®^ 488 anti-rabbit secondary antibody was obtained from Cell Signalling Technology (Beverly, MA, USA). pH: Merck indicator paper pH 1–14 (universal indicator). Chromatography: *Grace* silica gel (10–14 µm) with the indicated solvent system. Eluent mixtures are expressed as volume to volume (*v*/*v*) ratios. Thin-layer chromatography (TLC): *Merck* TLC plates silica gel 60 on Alox with the indicated solvent system and the spots were visualized with UV light (254 and 366 nm). UV-Vis spectra: Specord 250 Plus spectrophotometer (Analytik Jena, Jena, Germany); λ_max_ (logε) and λ_min_ (logε) in nm. Emission spectra were obtained on a *Perkin–Elmer* Luminescence spectrometer LS 50 B and were corrected for instrumental response using the provided correction factors. The excitation and emission slit width were set to 5.0 and 5.0 nm, respectively. IR spectra: SpectrumTwo FT-IR Spectrometer (Perkin–Elmer, Schwerzenbach, Switzerland) equipped with a Specac Golden Gate^TM^ ATR (attenuated total reflection) accessory, applied as neat samples, 1/λ in cm^–1^. ^1^H-NMR spectra in CDCl_3_ or DMSO-d_6_: *Bruker* AV-400 (400 MHz) or *Bruker* AV-500 (500 MHz). *δ* in ppm refer to the deuterated solvent CDCl_3_ (*δ* 7.26) or DMSO-d_6_ (*δ* 2.5) J in Hz. ^13^C-NMR spectra in CDCl_3_ or DMSO-d_6_: multiplicities from DEPT-135 and DEPT-90 experiments. The abbreviations for the peak multiplicities are as follows: s (singlet), d (doublet), dd (doublet of doublets), t (triplet), q (quartet), m (multiplet), and br (broad). The *Acquity* Waters UPLC (BEH C18 analytical column, 1.7 μm, 50 × 2.1 mm), equipped with a DAD detector and an auto sampler, was performed with a linear gradient of solvent A (distilled H_2_O containing 0.1% *v*/*v* TFA) and solvent B (CH_3_CN (Sigma-Aldrich HPLC-grade)); t = 0–0.5 min, 5% B; t = 0.51–4 min, from 95% A (5% B) to 0% A (100% B); t = 4–5 min, 0% A, 100% B. Detection was performed at 250 nm, 313 nm, and 640 nm. HR-EI-MS was performed on a Thermo DFS (ThermoFisher Scientific, Bremen, Germany) double-focusing magnetic sector mass spectrometer (geometry BE). Mass spectra were measured in electron impact (EI) mode at 70 eV, with a solid probe inlet, a source temperature of 200 °C, acceleration voltage of 5 kV, and resolution of 2500. The instrument was scanned between m/z 30 and 900 at scan rate of 2 s/decade in the magnetic scan mode. Perfluorokerosene (PFK, Fluorochem, Derbyshire, UK) was used for calibration. The UPLC–ESI–MS spectra of PSs were measured on an *Acquity* Waters UPLC system coupled to *Bruker* HCT^TM^ (Bremen, Germany) for the MS measurements, equipped with a DAD detector and an auto sampler using an *Acquity* UPLC BEH C18 analytical column (1.7 μm, 50 × 2.1 mm). The LC run (flow rate: 0.6 mL/min) was performed with a linear gradient of solvent A (distilled H_2_O containing 0.1% *v*/*v* formic acid) and solvent B (CH_3_CN (Sigma-Aldrich HPLC-grade)); t = 0–0.5 min, 5% B; t = 0.51–4 min, from 95% A (5% B) to 0% A (100% B); t = 4–5 min, 0% A, 100% B. UV-Vis detection was collected from 200 to 480 nm.

### 3.2. Syntheses

The phthalocyanines were isolated as green solids after column chromatography.

**LC31** (analogous to [9,40]):

Zn(OAc)_2_ (1.19 mmol, 0.22 g) and 4-(4-carboxyphenoxy)-phthalonitrile (**6**, 1.17 mmol, 0.31 g) were dissolved in 1-pentanol (17 mL) in a one-necked 100 mL round-bottom flask equipped with a reflux condenser. The mixture was stirred at 110 °C for 20 min. Then, the glycol-substituted nitrophthalonitrile (**3**, 3.69 mmol, 1.07 g) was added, followed by DBU (769 µL), and the colour of the mixture changed from orange to brown. The reaction mixture was then stirred at 160 °C under N_2_ atmosphere. After 48 h, the green reaction mixture was cooled down to room temperature and hexane (185 mL) was added. A green suspension/precipitate was formed, which was filtered off to obtain the crude product (**7**, 2.02 g). The crude product was purified by column chromatography on silica gel with chloroform and increasing amounts of MeOH. Analysis by UPLC showed a purity of 95%. NMR data were not obtained as **LC31** consists of a mixture of two isomers. UV-Vis (CHCl_3,_ λ_max_ nm, logε): 339 (6.01), 624 (5.82), 694 (6.55), 740 (5.69). (+)-UPLC-MS: 1199.7 (100, [*M* + H]^+^); calculated 1199.4, (100, [*M* + H]^+^). UPLC: R_t_ = 3.1.

**MLC31**:

To a suspension of 44.0 mg (0.037 mmol) of compound **LC31** in 3 mL of DMF, 11.6 mg (0.18 mmol) of *N*-methylmorpholine was added, followed by the addition of 9.0 mg (0.051 mmol) of 2-chloro-4,6-dimethoxy-1,3,5-triazine (CDMT) at 0 °C. The resulting mixture was stirred for 1.5 h until analysis by HPLC showed completeness of the activation. Dimethyl aminomalonate hydrochloride (10.8 mg, 0.051 mmol) was added, and the mixture was stirred at 25 °C until a complete conversion of MLC31 was observed by HPLC. A total of 10 mL of methylene chloride was then added to the reaction mixture together with 10 mL of deionized water, and the mixture was stirred further for 15 min. The layers were then separated, and the organic dark green layer was mixed with Celite before the solvent was evaporated to obtain a dry residue. Flash column chromatography (EtOAc/EtOH = 10:1 and CH_2_Cl_2_/CH_3_OH = 10:1) yielded **MLC31** (19 mg, 37% yield) as a dark green solid. The chemical purity assessed by NMR is 97%. M.p. > 300 °C, R_f_ (CH_2_Cl_2_/CH_3_OH = 10:1) = 0.14. UV−Vis (CDCl_3_, λ_max_ nm, logε): 694 (7.05), 626 (6.27), 336 (6.51). ^1^H-NMR (400 MHz; DMSO-d_6_): 11.99 (s, 1 H), 9.46–9.34 (m, 2 H), 9.13–8.90 (m, 4 H), 8.18–8.07 (m, 4 H), 7.94 (d, J = 8.2 Hz, 1 H), 7.84 (d, J = 8.2 Hz, 2 H), 7.74 (d, J = 7.6 Hz, 1 H), 7.49 (d, J = 8.3 Hz, 2 H), 5.38 (d, J = 7.6 Hz, 1 H), 5.19 (s, 3 H), 4.90 (s, 3 H), 4.40 (s, 3 H), 4.26 (q, J = 6.8 Hz, 4 H), 4.19 (s, 4 H), 4.07 (s, 3 H), 3.75 (s, 6 H), 3.60–3.52 (m, 7 H), 3.45 (s, 4 H), 3.42–3.37 (m, 3 H), 3.17 (s, 3 H), 3.11 (s, 6 H), 1.25 (t, J = 7.1 Hz, 6 H). ^13^C-NMR (101 MHz; DMSO-d_6_): 167.10, 130.77, 121.78, 118.63, 116.28, 109.99, 71.72, 71.65, 70.97, 70.60, 70.54, 70.29, 70.14, 70.00, 62.16, 58.46, 58.39, 57.06, 21.53, 14.39. ESI-MS: m/z 1356.8 [M + H]^+^, calculated 1356.4 [M + H]^+^. Retention time assessed by UPLC: R_t_ = 2.9 min.

**DMLC31Pt** (analogous to [78]):

**MLC31** (0.34 mmol, 0.91 mg, purity = 40%) was dissolved in toluene (870 µL) and acetonitrile (300 µL) in a 10 mL round-bottom flask equipped with a reflux condenser. Then, bis(tributyltin) oxide (0.16 mmol, 58 µL) was added and the reaction mixture was heated to 66 °C and stirred for 48 h. The solvent was concentrated with the rotary evaporator and then extracted with EtOAc (5 mL) and NaHCO_3_ (25%, 5 mL). The organic layer was removed, and the water layer was acidified with HCl (0.5 M, aq.) to a pH of 4. Then, EtOAc was added, and a precipitate was formed between the two layers. The precipitate was filtered and washed a few times with H_2_O and methanol, dissolved in DMF, and then dried under high vacuum (0.4 mbar) before letting it react with 2 eq. of KOH in H_2_O. *Cis*-[PtCl_2_(NH_3_)_2_] (0.249 g, 0.83 mmol) was added to a solution of Ag_2_SO_4_ (0.253 g, 0.81 mmol) in 40 mL H_2_O, and the mixture was stirred at 40 °C overnight in the dark. The mixture was then filtered to remove the formed AgCl and to get **8** dissolved in the filtrate. The solution obtained from the reaction between the deprotected **MLC31** and KOH in H_2_O was then added to the filtrate. The mixture was stirred at 30 °C overnight in the dark. The precipitate was filtered off, washed with MeOH and Et_2_O, and dried under vacuum to obtain a dark green crude product. The crude product was then purified with a reversed phase C_18_ modified silica gel column (MeOH:H_2_O starting at 1:4 and going up to 1:1) and dried on a lyophilizer to yield **DMLC31Pt** (0.0013 mmol, 2.0 mg, 19% yield). (+)-UPLC-MS: 1483.9 (100, [*M* + H]^+^); calculated 1483.4, (100, [M + H]^+^). UV−Vis (EtOH, λ_max_ nm): 693, 625, 337. ^1^H-NMR (400 MHz; DMSO-d_6_): 9.39–9.34 (m, 1 H); 9.10–9.08 (m, 1 H); 9.05–8.95 (m, 2 H); 8.90–8.82 (m, 2 H); 8.20–8.05 (m, 4 H); 7.85–7.74 (m, 4 H); 7.55–7.45 (m, 2 H); 5.20–5.18 (m, 2 H); 4.90 (s, 2 H); 4.64 (s, 8 H); 4.39 (s, 4 H); 4.19–4.18 (d, 2 H); 4.06 (s, 4 H); 3.75 (s, 6 H); 3.57–3.53 (m, 6 H); 3.44–3.43 (m, 4 H); 3.17–3.15 (m, 3 H); 3.12–3.12 (m, 6 H). ^195^Pt-NMR (107 MHz; DMF-d_7_): −1760, −2102. HR-MS: m/z 1278.37107 [*M* + Na]^+^, calculated 1278.37329 [*M* + Na]^+^. Retention time assessed by UPLC: R_t_ = 2.6 min.

## 4. Conclusions

A stepwise functionalization of the peripheral moieties on highly aromatic Pc-based PSs was investigated. Follow-up optical microscopic and cytotoxic studies revealed the impact of this modification and that the presence of a malonic ester functionality is indispensable to confer photocytotoxicity in this Pc-based agent series. After identification of the best performing mitochondria-targeting anticancer PDT agent, **MLC31** (IC_50_ = 157 nM in the cisplatin resistant A2780/CP70 cell line upon NIR light activation and IC_50_ = 117 μM in dark, p.i.~750), an effective cytotoxic activity upon light activation was demonstrated. A preliminarily investigation of its behavior in cells and its mechanism of action showed how the functional groups affect the distribution coefficient (log*P*) and therefore the cellular uptake. Additionally, how **MLC31** disrupts the Δ*Ψ*_m_ and induces apoptosis upon activation by light was investigated. Surprisingly, **MLC31** showed high cytotoxicity under hypoxic conditions, which is due to its mitochondria-targeting property. In conclusion, the amphiprotic property of **MLC31** dramatically enhances the intercalation with the mitochondrial membrane, thus improving the mitochondria-targeting uptake. Such an asymmetric design with both hydrophilic and lipophilic peripheral groups may serve as an effective way to improve the PDT efficiency of highly aromatic PSs for NIR light-mediated cancer therapy.

## Figures and Tables

**Table 1:** Photophysical properties of **LC31** and **MLC31** in PBS, DMSO and MeOH.

	B-Band (nm)	Q-Band (nm)	*ϵ* of the Q-Band (M^−1^ cm^−1^)			λ_em_ (nm)	*τ*_F_ (ns) ^a^	*Stokes* shift (nm) ^a^	Φ_∆_ ^b^
	DMSO	DMSO	PBS	DMSO	MeOH	DMSO			
**LC31**	338	696	37,400	114,800	105,000	704	2	8	0.49
**MLC31**	345	694	57,900	206,900	145,800	707	3	13	0.84
**DMLC31Pt**	350	693	19,500	79,000	31,000	697	-	4	0.39

^a^ in MeOH. ^b^ Φ_MB_ = 0.52 in MeOH [44]. Light with a wavelength cutoff below 600 nm with a power intensity of 5.8 mW cm^−2^ was applied with a white light projector. The total light dose equals to 6.96 J cm^−2^.

**Table 2:** Cytotoxic activity data (IC_50_) for Pc-based agents **LC31**, **MLC31**, **DMLC31Pt**, and cisplatin against human cervical cancer (HeLa), human ovarian carcinoma (A2780), its cisplatin resistant variant (A2780/CP70), and non-cancerous fibroblast (MRC-5) cell lines.

Comp.	HeLa (μM)			A2780 (μM)			A2780/CP70 (μM)			MRC-5 (μM)		
	Dark ^a^	Light ^b^	p.i. ^c^	Dark ^a^	Light ^b^	p.i. ^c^	Dark ^a^	Light ^b^	p.i. ^c^	Dark ^a^	Light ^b^	p.i. ^c^
**LC31**	14.7	0.426	**35**	31.0	0.211	**147**	69.5	1.2	**58**	48.1	1.1	**44**
**MLC31**	5.2	0.009	**578**	44.0	0.018	**2444**	117.5	0.157	**748**	12.8	0.019	**674**
**DMLC31Pt**	8.0	0.775	**10**	25.0	0.199	**126**	88.7	0.773	**115**	61.5	0.845	**73**
cisplatin	1.3	1.7	**0.76**	2.8	4.0	**0.7**	102.3	128.8	**0.79**	5.4	4.5	**1.2**

^a^ Cells were incubated with the indicated compounds in the dark for 4 h. ^b^ Cells were incubated with the indicated compounds for 4 h in the dark and then irradiated with a white light projector (filter cutoff below 600 nm, light intensity 5.8 mW cm^−2^, light dose 6.96 J cm^−2^). ^c^ p.i. = phototoxicity index, the ratio of the IC_50_ values in the dark to those obtained after light irradiation.

**Table 3:** Comparison of toxicities in the dark and upon light irradiation of Pcs with a neutrally charged central unit against the human cervical cancer (HeLa) cell line. Only the best compound from each publication is taken.

Comp.	Dark Toxicity [μM]	Light Toxicity [μM]	p.i.	Wavelength [nm]	Fluence [J cm^−2^]	Ref.
**MLC31**	5.2	0.009	578	λ > 600 ^a^	6.96	This work
(SiPc)((OC_2_H_4_)_22_OH)_2_	>3	0.3	>10	690 ^b^	2	[56]
SiPc(R-Biotin)_2_	>10	0.4	>25	690 ^b^	2	[57]
ZnPc(branchedPEG)_3_ biotin	>5	1.5	>3.3	690 ^b^	2	[58]
ZnPc(I_3_)R-Biotin	>10	2.2	>4.6	690 ^b^	2	[59]
[(SiPc)(RNEt_2_Me)_2_]^2+^	>10	1.5	>6.7	690 ^b^	2	[60]
[ZnPc]^8+^	>100	0.04	2500	740 ^c^	15	[61]
[ZnPc-(R(pyMe)_4_)_4_]^16+^	675	0.48	1409	λ > 570 ^d^	11.2	[62]
[ZnPc-(R’imidazolium_4_)_4_]^16+^	395	0.037	10675	λ > 570 ^d^	11.2	[63]

^a^ Halogen lamp, ^b^ Lumacare (LC-122 fiber-optic probes), ^c^ LED, ^d^ Xe lamp.

**Table 4:** Comparison of the IC_50_ values (μM) of **LC31, MLC31**, and **DMLC31Pt** under hypoxic or normoxic conditions.

	18.6% O_2_			0.2% O_2_		
	Dark	Light ^a^	p.i.	Dark	Light ^a^	p.i.
**LC31**	>20	0.169	>118	>20	0.177	>113
**MLC31**	14.8	0.051	290	>20	**0.061**	**328**
**DMLC31Pt**	16.7	7.0	2.4	>20	8.1	>2.5

^a^ Irradiation experiment with an LED 660 nm light source, light intensity = 9 mW cm^−2^, light dose = 10.8 J cm^−2^.

## Data Availability

Additional data are available as the Supplementary Information.

## Supplementary Materials

The supporting information can be downloaded at: https://www.mdpi.com/article/10.3390/ijms23179525/s1. References [9,26,33,40,43,66,67,79,80,81,82,83,84,85,86,87,88,89] are cited in the supplementary materials.
