# Supplementary material for: Low-Dose Near-Infrared Light-Activated Mitochondria-Targeting Photosensitizers for PDT Cancer Therapy
Source: Int J Mol Sci. 2022 Aug 23;23(17):9525. doi: 10.3390/ijms23179525 (PMC9455738; doi:10.3390/ijms23179525)
Supplement: Supplementary file 1 [file ijms-23-09525-s001.zip › ijms-1861401-supplementary.pdf]

Supporting Information

Low Dose Near-Infrared Light Activated Mitochondria-Targeting Photosensitizers for PDT Cancer Therapy

Wenyu Wu Klingler,[1,2] Nadine Giger,[1] Lukas Schneider,[1] Vipin Babu,[1] Christiane König,[3] Patrick Spielmann,[4] Roland H. Wenger,[4] Stefano Ferrari,[3]* Bernhard Spingler[1]*

1 Department of Chemistry, University of Zurich, Winterthurerstrasse 190, 8057 Zurich, Switzerland. Web: https://www.chem.uzh.ch/en/research/groups/spingler.html.

2 Laboratory for Advanced Fibers, Empa Swiss Federal Laboratories for Materials Science and Technology, Lerchenfeldstrasse 5, 9014 St. Gallen, Switzerland

3 Institute of Molecular Cancer Research, University of Zurich, Winterthurerstrasse 190, 8057 Zurich, Switzerland.

4 Institute of Physiology, University of Zurich, Winterthurerstrasse 190, 8057, Zurich, Switzerland.

* Correspondence: sferrari@imcr.uzh.ch and spingler@chem.uzh.ch; Tel.: +41 44 635 46 56

Crystallographic methods

Crystallographic data were collected at 160.0(1) K on a *Rigaku-Oxford* Diffraction XtaLAB Synergy-S dual source diffractometer. This is a kappa-axis four-circle goniometer with a *Dectris* Pilatus3 R 200K HPC (Hybrid Photon Counting) detector and Cu and Mo PhotonJet microfocus X-ray sources. Suitable crystals were covered with oil (Infineum V8512, formerly known as Paratone N), placed on a nylon loop that is mounted on a CrystalCap Magnetic™ pin (*Hampton Research*) and immediately transferred to the diffractometer. The program suite CrysAlis^{Pro} was used for data collection, numerical and multi-scan absorption correction as well as data reduction.[1] The structures were solved with the dual-space algorithm using *SHELXT*[2] and was refined by full-matrix least-squares methods on F^2 with *SHELXL-2018*[3] using the *Olex2* GUI.[4] The graphical output was produced with the help of the program Mercury.[5] CCDC 2151600 contains the supplementary crystallographic data for this paper. These data are provided free of charge by The Cambridge Crystallographic Data Centre *via* www.ccdc.cam.ac.uk/structures.

Synthesis

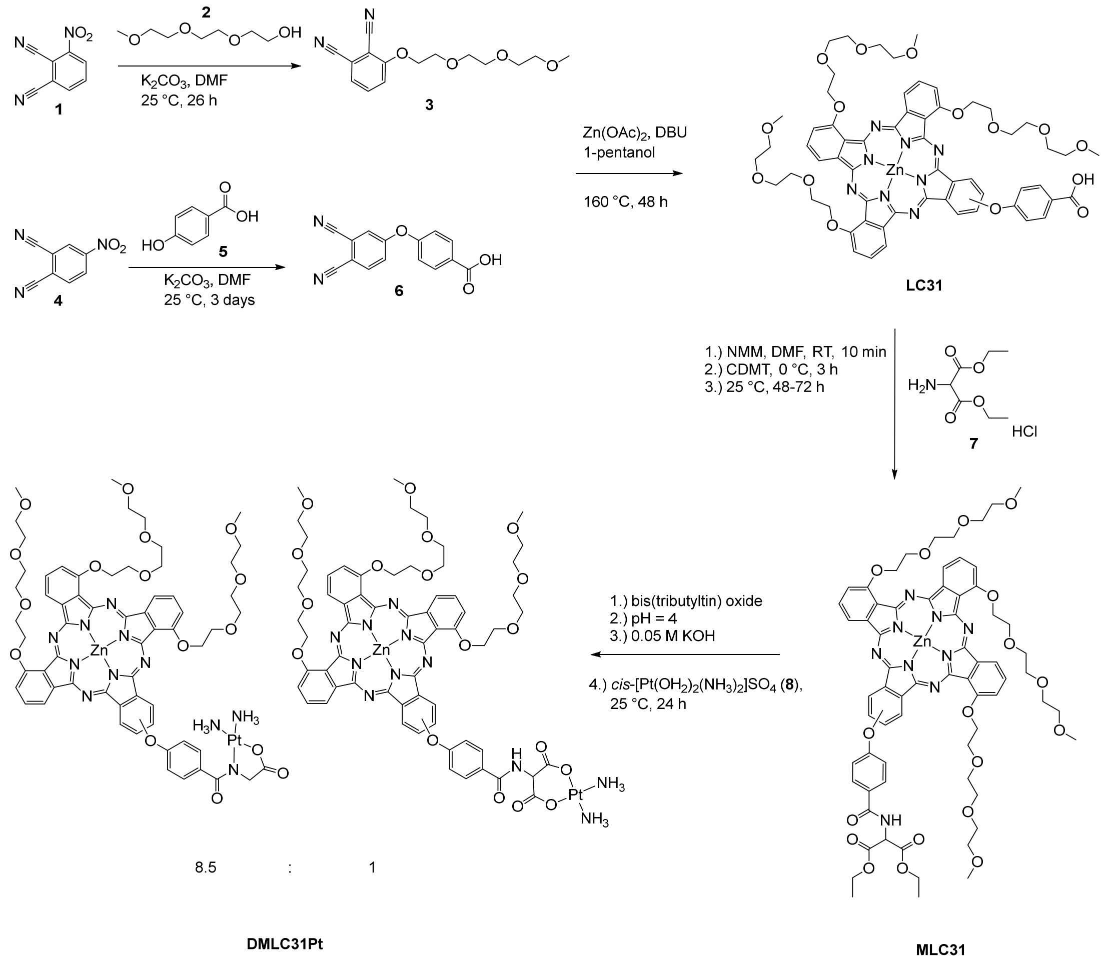

Scheme S1. Synthesis route yielding **LC31**, **MLC31**, and **DMLC31Pt**.

The phthalonitriles **3**, and **6** were applied in a macrocyclization reaction in *n*-pentanol in the presence of zinc(II) acetate and 1,8-diazabicyclo[5.4.0]undec-7-ene (DBU) as a base to give a novel A_3B

disubstituted non-symmetrical zinc(II) Pc **LC31** (Scheme S1) according to reported procedures.[6,7] The macrocyclic and platinated Pc products were isolated as green solids after column chromatography.

3 ref. [8]:

3-Nitrophthalonitrile (**1**, 29.19 mmol, 5.05 g) was dissolved in DMF (50.0 mL) in a 250 mL round bottom flask, then triethyleneglycol monomethylether (**2**, 64.73 mmol, 10.63 g) was added. After that K_2CO_3 (130.86 mmol, 18.09 g) was added and the reaction was stirred under an N_2 atmosphere. A change in colour from light yellow to orange could be observed. After 1 h the colour changed from orange to dark red. The reaction progression was controlled by UPLC. After 26 h of stirring at 25 °C the reaction was finished and the mixture was poured into 1700 mL of ice water and left overnight. A white precipitate could be observed. The white precipitate was filtered off, washed multiple times with water and dried on a lyophilizer overnight. The precipitate was recrystallized from MeOH (10 mL) and further purified by column chromatography on silica gel with (EtOAc/hexane = 3:1, followed by pure EtOAc) to afford (**3**, 14.75 mmol, 4.28 g, 51% yield, >95% purity).

(+)-UPLC-MS: 291.1 (100, $[M + H]^+$); calculated 291.1, (100, $[M + H]^+$). IR: 3080*w* (Aryl-H), 2900*w* (O-CH_3), 2228*w* (C≡N), 1580*m* (arom. C), 1474*m* (CH_2), 1351*m*, 1290*s* (C-O-C), 1194*w*, 1137*w*, 1104*m*, 1092*s*, 1057*s* (C-O-C), 1027*m*, 996*m*, 849*m*, 806*m*, 736*w*, 689*w*. ^{1}H-NMR ($CHCl_3$): 7.64 – 7.60 (*m*, 1 H, OCCHC*H*); 7.36 – 7.30 (*m*, 2 H, OCCHCHC*H* and OCC*H*); 4.31 (*t*, *J* = 4.7, 2 H, NCCCOC*H₂*); 3.93 (*t*, *J* = 4.7, 2 H, NCCCOCH$_2$C*H*$_2$); 3.77 – 3.75 (*m*, 2 H, NCCCO(CH_2)$_2$O(CH_2)$_2$OCH$_2$C*H*$_2$); 3.68 – 3.63 (*m*, 4 H, NCCCO(CH_2)$_2$OC*H*$_2$ or NCCCO(CH_2)$_2$OCHC*H*$_2$ or NCCCO(CH_2)$_2$O(CH_2)$_2$OC*H*$_2$); 3.56 – 3.53 (*m*, 2 H, NCCCO(CH_2)$_2$OC*H*$_2$ or NCCCO(CH_2)$_2$OCHC*H*$_2$ or NCCCO(CH_2)$_2$O(CH_2)$_2$OC*H*$_2$); 3.37 (*s*, 3 H, CH_3). ^{13}C-NMR ($CHCl_3$): 161.5 (*s*, NCC*C*O); 134.6 (*d*, OCCH*C*H); 125.4 (*d*, OCCHCH*C*H); 117.5 (*d*, OC*C*H); 117.0 (*s*, O(CH_2)$_3$*C*CN); 115.3 (*s*, O(CH_2)$_3$C*C*N); 113.1 (*s*, OCC*C*N); 105.2 (*s*, OC*C*CN); 72.0 (*t*, CH_3O*C*H$_2$); 71.2 (*t*, CH_3O(CH_2)$_2$OCH$_2$*C*H$_2$); 70.7 (*t*, CH_3O(CH_2)$_2$O*C*H$_2$CH$_2$); 70.6 (*t*, CH_3OCH$_2$*C*H$_2$); 69.8 (*t*, CH_3O(CH_2)$_2$O(CH_2)$_2$O*C*H$_2$CH$_2$); 69.3 (*t*, CH_3O(CH_2)$_2$O(CH_2)$_2$OCH$_2$*C*H$_2$); 59.1 (*s*, CH_3).

6 ref. [9]:

DMF (50 mL) was prepared in a 250 mL one-necked round bottom flask. Then 4-nitrophthalonitrile (**4**, 26.26 mmol, 4.54 g), 4-hydroxybenzoic acid (**5**, 43.45 mmol, 6.00 g) and K_2CO_3 (125.71 mmol, 5.99 g) were added and the colour of the reaction mixture turned light yellow. The mixture was then stirred at 25 °C for 3 days. After the reaction was complete, distilled H_2O (700 mL) was added to the dark brown suspension and the pH of the mixture was adjusted to 1 using concentrated HCl (aq.). The colour changed from dark brown to light brown and the formed light brown precipitate was filtered off, washed three times with H_2O and dried *in vacuo* (25°C, 1 mbar) to afford the crude product (16.621 g). The crude product was recrystallized from MeOH (120 mL) to afford **6** as a light brown solid (2.15 mmol, 5.68 g, 82%, >95% purity).

(+)-UPLC-MS: 332.8 (100, $[M + HCl + MeOH + H]^+$); calculated 233.6, (100, $[M + HCl + MeOH + H]^+$). IR: 3039*w* (arom. H), 2552*w*, 2236*w* (C≡N), 1678*s* (Aryl-COOH), 1584*s*, 1486*s*, 1428*s*, 1293*s*, 1271*s* (O-H), 1247*s*, 1212*s* (C-O), 1164*s* (Aryl-O-Aryl), 1112*w*, 950*s*, 883*m*, 845*s*, 774*s*, 692.4*w*, 656*w*. ^{1}H-NMR ((CD_3)$_2$SO): 8.16 (*d*, *J* = 8.8, 2 H, C*H*C(COOH)C*H*); 8.10 (*d*, *J* = 8.7, 1 H, OCCHC*H*CCN); 7.78 (*d*, *J* = 2.5, 1 H, OCC*H*CCN); 7.58 (*dd*, *J* = 8.7, 2.5, 1 H, OCC*H*CHCCN), 7.33 (*d*, *J* = 8.7, 2 H, OC(C*H*)$_2$). ^{13}C-NMR ((CD_3)$_2$SO): 166.8 (*s*, *C*OOH); 161.4 (*s*, O*C*(CH)$_2$CCOOH); 159.8 (*s* O*C*CHCCN); 137.0 (*d*, OCCH*C*HCCN); 133.2 (*d*, 2 x *C*HCCOOH); 128.6 (*s*, *C*COOH); 124.1 (*d*, OC*C*HCHCCN); 124.0 (*d*, OC*C*HCCN); 120.7 (*d*, 2 x OC*C*HCHCCOOH); 118.5 (*s*, OCCH*C*CN); 116.2 (*s*, OC(CH)$_2$*C*CN); 115.9 (*s*, OCCHC*C*N); 110.8 (*s*,

OC(CH)$_2$*C*CN).

From the solution in DMSO-d$_6$ in an NMR tube of the column fractions of **LC31** containing the symmetric Pc **LC4** as well,[8,10] crystals of **LC4** were obtained (Figure S1). Three of the PEG groups are disordered, one of them to such an extent that the methyl group and the minor component of the disorder could not be found. One DMSO molecule is coordinated to the zinc center.

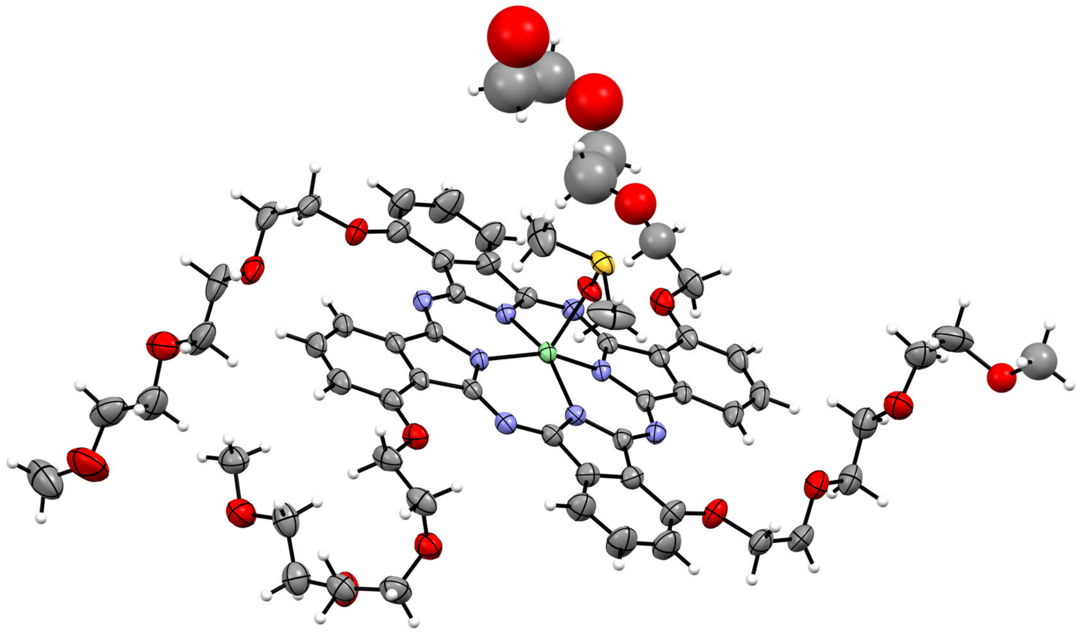

Figure S1: Ellipsoidal representation of **LC4**. Ellipsoids are shown at 50% probability. Only the major components of the disordered PEG groups are shown. One methyl group could not be found in the crystal structure.

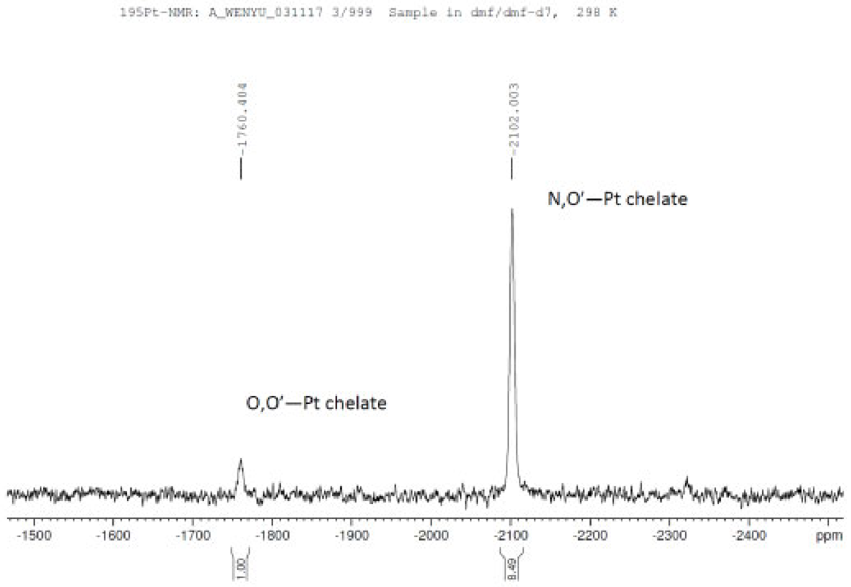

Figure S2. ^{195}Pt-NMR of the **DMLC31Pt** in DMF/DMF-d_7 = 4:1 (v/v).

Table S1. Comparison of theoretical and experimental chemical shifts of the ^{195}Pt-NMR of **DMLC31Pt** and reported Pt^{II} complexes.

Complex coordination type	Theoretical chemical shift [ppm][11]	Measured chemical shift [ppm]
**(N,O)**	~ -2100	-2102
**(O,O')**	-1700 – -1750	-1760

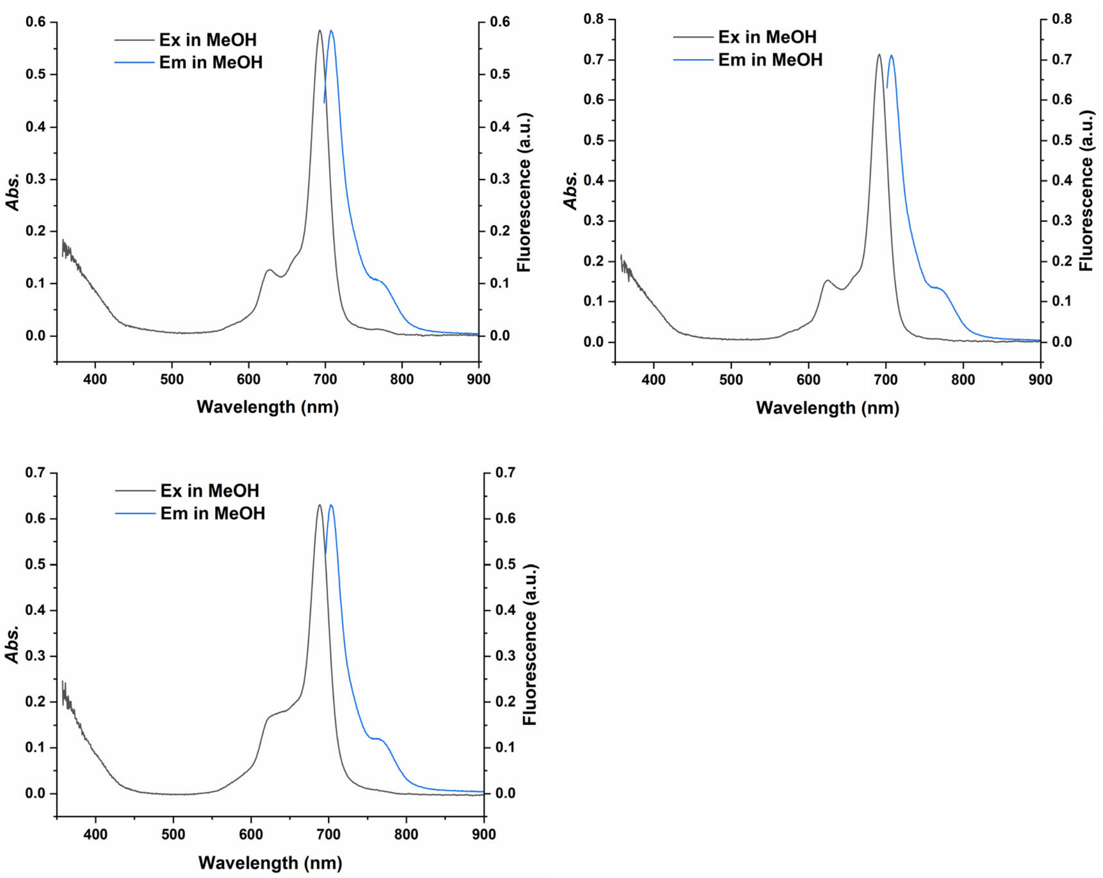

Figure S3: UV-Vis absorption spectra of top left) **LC31**, top right) **MLC31**, and bottom) **DMLC31Pt** in MeOH, and the corresponding normalized emissions.

${}^{1}O_2$ *Quantum Yields (Φ_Δ) in methanol and ROS generation in aqueous environment*

${}^{1}O_2$ can promote the level of cytotoxic ROS in cancer cells and then cause oxidative damage to cancer cells, leading to a crucial role in PDT. The Φ_Δ of **LC31**, **MLC31** and **DMLC31Pt** at 4 μM concentration were determined by an 1,3-diphenylisobenzofuran (DPBF) bleaching assay relative to methylene blue (MB) that has a Φ_Δ of 0.52 in aerated MeOH, according to the literature.[12] All samples were exposed to a xenon lamp equipped with a 600 nm cut-off filter (light intensity = 20 mW cm^{-2}), if not mentioned otherwise. The bleaching rate of 50 μM DPBF was followed at 410 nm. The slope (*k*) of the plot of bleached absorption (−A) at 410 nm vs. irradiation time is proportional to the rate of ${}^{1}O_2$ production. This method is based upon bleaching of DPBF as induced by the reaction of ${}^{1}O_2$ which results in the formation of an endoperoxide intermediate.

Air-saturated MeOH was obtained by bubbling air into MeOH for 5 min. The photooxidation of DPBF was monitored between zero s to few min depending on the efficiency of the PS. The Φ_Δ can then be calculated with a relative method by comparing the Φ_Δ of the corresponding PS to the one of MB as the reference. The Φ_Δ were calculated according to equation (**2**):

$$\Phi_\Delta = \Phi_{(MB)} \times ((k_{(Pc)} \times A_{(MB)}) / (k_{(MB)} \times A_{(Pc)})) \qquad \mathbf{2}$$

where *k* is the slope of the difference in the change of absorbance of DPBF at 410 nm vs. the irradiation time, and *A* is the absorption intensity at the irradiation wavelength.

Light activation experiments were performed using a *Reflecta* Diamator AF 2006 IR Hobby Line 250W projector with a 600 nm long pass cut-off filter (60 mm square, 1 mm thickness).

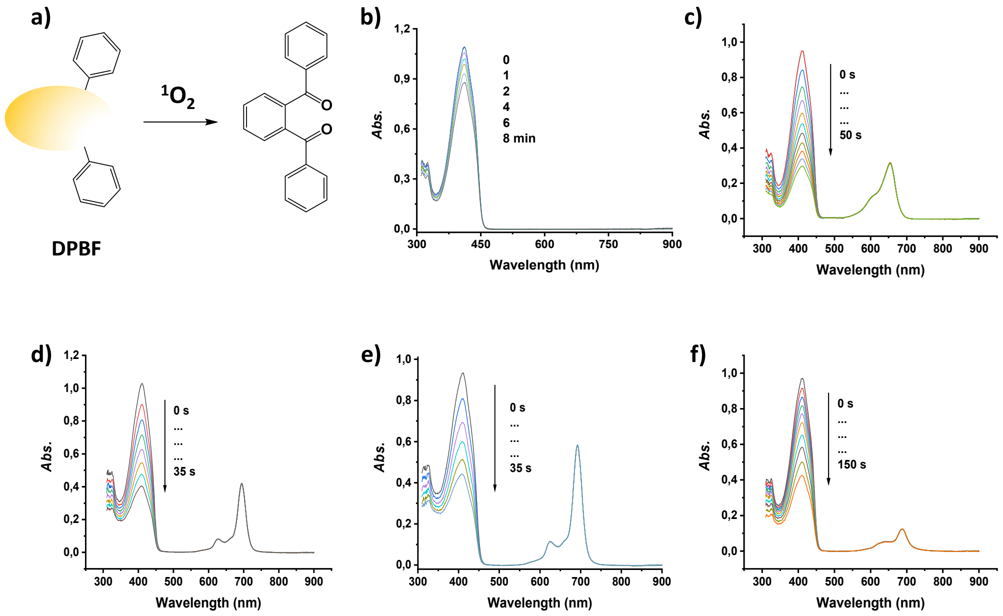

Figure S4. a) Reaction mechanism of 1,3-diphenylisobenzofuran (DPBF) with ${}^{1}O_2$. (b-d) Change of UV-Vis absorption spectra after photoirradiation. b) DPBF alone, c) **MB**, d) **LC31**, e) **MLC31**, and f) **DMLC31Pt** (concentration = 4 μM) in MeOH using DPBF (50 μM) as the ${}^{1}O_2$ probe.

*Generation and detection of hydroxyl radicals (•OH) upon light irradiation of compounds **LC31, MLC31** and **DMLC31Pt***

The fluorescein-derived hydroxyl radical (•OH) sensor aminophenyl fluorescein (APF) was synthesized as described in literature[13] and used as a turn-on sensor to visualize the •OH radicals production by the PSs through the oxidation of the non-fluorescent APF, leading to the fluorescent product fluorescein.[14] A *Varian* Cary Eclipse fluorescence spectrometer was used to detect the fluorescence of the emerging fluorescein while using the following settings: Excitation at 492 nm, collection of the emission at 525 nm, slit width: 2.5 nm for excitation and emission, averaging time: 1 second. The cuvette was kept at a constant temperature of 25 °C while stirring.

As positive control, the *Fenton*'s reagent was applied to oxidize the APF. For this purpose, a freshly prepared solution of 5 µM APF (from a stock solution of APF in DMF) and 300 µM ammonium iron(II) sulfate (FAS, from a stock solution of FAS in H_2O) in PBS (3 mL total volume) in a cuvette was used as a blank and measured for 30 s to ensure no fluorescence signal is detectable at 525 nm. Then, after 30 s, H_2O_2 was added during the measurement in a way that the final H_2O_2 concentration reached 300 µM and the solution was measured for another 30 s to visualize the increase in fluorescence, showing the reactivity of the synthesized APF towards the produced •OH radicals (see reference [15] for an image of the control tests).

To show the generation of •OH radicals by **LC31**, **MLC31** and **DMLC31Pt** upon light irradiation, 1 mM stock solutions of **LC31**, **MLC31** and **DMLC31Pt** in DMF were prepared. Then, 30 µL of the stock solution were 1000-fold diluted with 3 mL of a PBS solution containing 5 µM APF for the experiment. The fluorescence intensity of the solution was measured at 525 nm for 1 min, before the cuvette was irradiated for 1 min with light of 690 nm at a distance of 1 cm from the LED light source while stirring (light intensity at the distance applied: 8 mW cm^{-2}) and the fluorescence intensity measured again. This process was repeated in 1 min irradiation steps until 5 min of total irradiation time were reached. The mean and the standard deviation of the fluorescence signal for every measurement were determined and plotted below, showing the relative •OH radicals production rates of **LC31**, **MLC31** and **DMLC31Pt** (Figure S5).

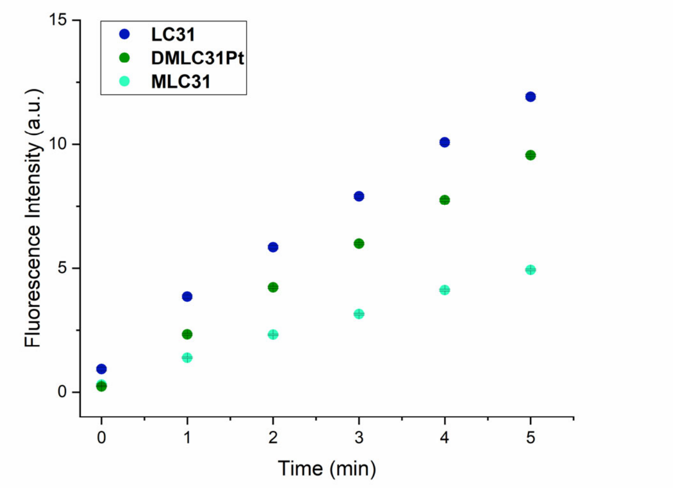

Figure S5: Fluorescence of APF as a function of LED light irradiation at 690 nm.

Determination of the Partition Coefficients ($P_{O/W}$)

The distribution coefficient of each complex was experimentally determined using the *shake-flask* method.[16,17] Briefly, 5 mL of a 10 µM solution from each Pc derivative was prepared in *n*-octanol. The UV–Vis spectrum of solution was sampled. Then, water (5 mL) was added to solution and the container was stirred for 30 min. The centrifugation (5 min at 5000 rpm) enabled a phase separation and the organic phase was sampled again. The partition coefficient was obtained from the difference in the Pc absorption intensity (around 695 nm) in both stages. At least three independent measures were performed and the corresponding $P_{O/W}$ value was taken as the overall average.

Cell Culture

Human cervical carcinoma cells (HeLa) were cultured in DMEM (*Gibco*) supplemented with 5% fetal calf serum (FCS, *Gibco*), 100 U/mL penicillin, 100 µg/mL streptomycin. The normal human fetal lung fibroblast cell line (MRC-5) was grown in MEM medium (*Gibco*) supplemented with 10% FCS (*Gibco*), penicillin (100 U/mL), and streptomycin (100 µg/mL). The human ovarian carcinoma A2780 cell line and a cisplatin-resistant subline CP70 were cultured in RPMI-1640 medium (*Gibco*) supplemented with 10% FCS (*Gibco*), penicillin (100 U/mL), and streptomycin (100 µg/mL). The cells were cultured at 37 °C and in 5% CO_2 humidified atmosphere.

Cytotoxicity Determination

The cytotoxicity of the PSs in the dark and upon light irradiation was evaluated in several cell lines, using a resazurin-based fluorometric cell viability assay (*Sigma-Aldrich*, Darmstadt, Germany). Stock solutions of the Pc agents (5 mM) were prepared in DMF and stored in the dark. The stock solutions were further diluted with complete medium to the desired working concentrations. For an experiment, 100 µL aliquots of cells in growth medium were seeded in 96-well plates (density of 2.5×10^3 cells/well for HeLa, 4×10^3 cells/well for A2780, and 1.5×10^3 cells/well for MRC-5 cells) and incubated at 37 °C, 5% CO_2 and 100% humidity. After 24 h of incubation, cells were treated with different concentrations (nanomolar to micromolar scale, 100 µL final well volume) of the test compounds and incubated for 4 h in the dark. For light irradiation experiments, the cell medium containing the corresponding PS was replaced by fresh medium after 4 h of incubation. Each sample was placed under the red cut-off filter at a distance of 20 cm from the projector. Then, cells were exposed to light (white light projector with a 600 nm cut-off filter) for 20 min (light intensity = 5.8 mW cm^{-2} for 20 min, light dose = 6.96 J cm^{-2}), followed by incubation for additional 72 h. Thereafter, the medium was removed, 100 µL of freshly prepared resazurin-containing complete medium (0.2 mg/mL final concentration) was added and the cells were incubated at 37 °C for additional 2 to 4 h. At the end of the incubation period, fluorescence of the highly red fluorescent resorufin product (λ_{ex} = 540 nm, λ_{em} = 590 nm) was quantified using a SpectraMax M5 microplate reader (*Molecular Device*, San Jose, USA). The reported cytotoxicity data is an average of at least two independent experiments, with triplicate determinations for each drug concentration. The final DMSO concentration in the wells was less than 0.5% (v/v). Control experiments with cells treated with the same concentration of DMSO in culture medium showed no cytotoxic effect (data not shown).

The cytotoxicity determination under hypoxic conditions was carried out following the same procedure as above, except that after addition of the PSs the cell dishes were placed in a hypoxia workstation

(InvivoO_2 400, *Baker Ruskinn Technology*, Ltd., Bridgend, UK) with a O_2 concentration of 0.2%. After 4 h of cellular uptake, the medium was replaced by hypoxically preconditioned medium and the cells were irradiated within the hypoxia workstation. Later on, cells were placed back into a normoxic incubator for further process.

Immunostaining

HeLa cells were seeded at a density of 3 x 10^5 cells/mL in a 6 cm dish containing cover slips. The next day, cells were treated for 4 h with **MLC31**, and **DMLC31Pt** (200 nM), the medium was replaced and cells were irradiated with a halogen light projector (1.29 J cm^{-2}). After treatment, cells were placed back into the incubator for 16 h. Cells were then fixed for 15 min at RT in 4% formaldehyde, permeabilized in 0.1% Triton X-100 for 5 min at 4 °C, blocked in 3% milk/PBS and incubated over night at 4 °C with anti-γH2AX antibodies. After washing in 3% milk/PBS, cells were treated with Alexa Fluor 488 goat-anti rabbit antibodies (1:1000) for 1 h at 37 °C. After washing in PBS and rinsing in double deionized H_2O, coverslips were mounted on slides with DAPI containing mounting solution (*Vectashield*) and visualized with a CLSM SP5 microscope.

In Vitro Fluorescence Evaluation and Mitochondrial Staining.

To examine the intracellular localization of **LC31**, **MLC31** and **DMLC31Pt**, HeLa cells were seeded and allowed to grow to 2.0 x 10^6 per dish in 35-mm glass bottom confocal dishes (SPL). Cells were incubated with medium containing **LC31**, **MLC31**, and **DMLC31Pt** (5 μM, respectively) for 4 h and then washed three times with PBS. Co-localization of the Pc-based agents with the mitochondria was examined by means of mitochondria-specific dyes, MitoTracker Green/Deep Red FM (λ_{ex} / λ_{em} = 490 / 516 nm and λ_{ex} / λ_{em} = 644 / 665 nm, *Molecular Probes*), for live-cell and fixed cell imaging, respectively. Briefly, a 1 mM MitoTracker stock solution made in DMSO was diluted to a 10 μM working concentration in cell medium (DMEM, 5% FCS). The staining of mitochondria was then accomplished by adding a 50 nM final concentration of the MitoTracker dyes to the culture medium for the last 45 min of PS incubation. For live-cell imaging, cells were washed with PBS and directly imaged by confocal microscopy. For fixed-cell imaging, the medium was removed, and cells were fixed in 4% formaldehyde solution before being mounted on slides for viewing by confocal microscopy. The intracellular localization of **LC31**, **MLC31**, and **DMLC31Pt** were inferred by imaging the overlapped fluorescence from the test agent and the dye in question using a Leica SP8 laser scanning confocal microscope with λ_{ex} = 670 nm and λ_{em} =700-750 nm. The overlapping fluorescence intensity was quantified and analyzed using the ImageJ free-software package[18] (*National Institutes of Health*, USA, https://imagej.nih.gov/ij/).

Flow cytometry analysis

HeLa cells were incubated with PSs (0 nM, 500 nM, 2 μM, and 8 μM) for 4 h. Suspensions of HeLa cells treated with PSs were washed twice in PBS and centrifuged at 130 g for 6 min to remove the medium and the Pc-based agents. The cellular uptake was detected in the Alexa Fluor 700 channel (λ_{ex} = 638 nm; λ_{em} = 700/710 nm). A total of 10 000 cells were collected for each sample by the flow cytometer CyAn ADP 9 and analyzed with the Summit 4.3 software. Non-viable cells were excluded from the analysis. The data is represented as mean ± SEM.

Detection of Intracellular ROS Levels

2 x 10^5 HeLa cells in 2 mL DMEM supplemented with 5% FBS were seeded on 60 mm dishes. The following day, cells were treated with 1 µM of the respective PS for 4 hours. Following incubation, the cells were washed with PBS and the medium was subsequently replaced with 2 mL DMEM. Cells were irradiated for 20 min with a halogen lamp, as described in the photocytotoxicity assay procedure. After irradiation, H_2DCF-DA was added to the samples to achieve a final concentration of 50 µM and they were incubated for 30 min at 37 °C. Cells were then washed twice with PBS, collected by trypsinisation and suspended in 1 mL PBS containing 1% FBS. The samples were analysed on an Attune NxT Cytometer (*Thermo Fisher Scientific*) using the BL1 channel (λ_{ex} = 488 nm, λ_{em} = 515 nm) to measure the DCF fluorescence. Results are expressed as mean percentage of ROS levels over the cell population, obtained from three independent experiments.

Evaluation of Mitochondrial Membrane Potential ($\Delta\Psi_m$).

The assay is based on the use of a cationic hydrophobic mitochondrial potential dye which accumulates in normal mitochondria, while in the treated cells, a $\Delta\Psi_m$ collapse results in decreased fluorescence, indicating apoptosis and the releasing of cyt c into the cytosol. Detection of the loss of the $\Delta\Psi_m$ in HeLa cells was executed using the Mitochondria Membrane Potential Kit (MAK147, λ_{ex} = 540 nm; λ_{em} = 590 nm, *Sigma–Aldrich*). HeLa cells were seeded in 6-well plates at a density of 4 x 10^5 cells one day before treatment and incubated for 2 h with 500 nM **MLC31** at 37 °C. Following this, the cells were treated with trypsin and resuspended in 5 mL of complete medium. The cell suspension was then stained according to the instructions. After 48 h of incubation, the cell medium was removed and the Loading Dye Solution was added. Cells were incubated in 5% CO_2 at 37 °C for 30 min. Afterwards, 50 µL of Assay Buffer B was added to each well and cells were incubated for 30 min. The fluorescence intensity was measured at λ_{ex} = 540 and λ_{em} = 590 nm.

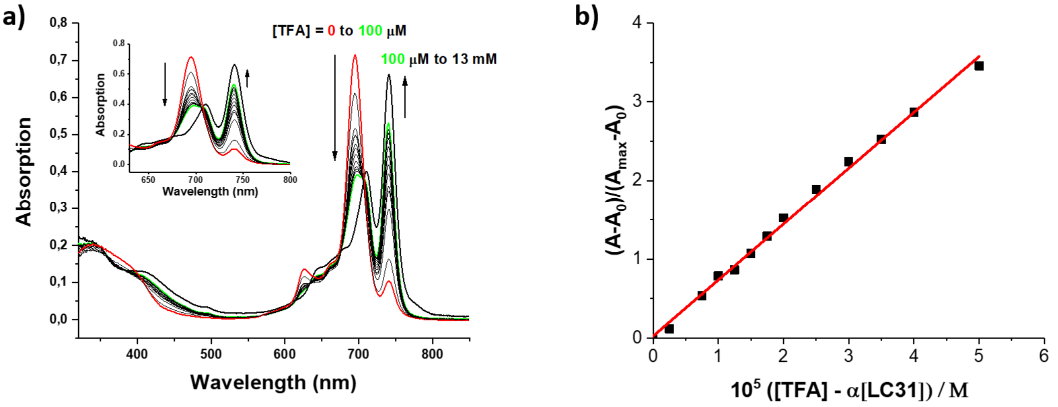

Figure S6. a) UV-Vis spectral changes accompanying the protonation of **LC31** in $CHCl_3$ upon addition of TFA to the solution of **LC31** (20 µM). b) Plots showing the one-step equilibrium of the reaction of **LC31** with TFA in $CHCl_3$ at room temperature.

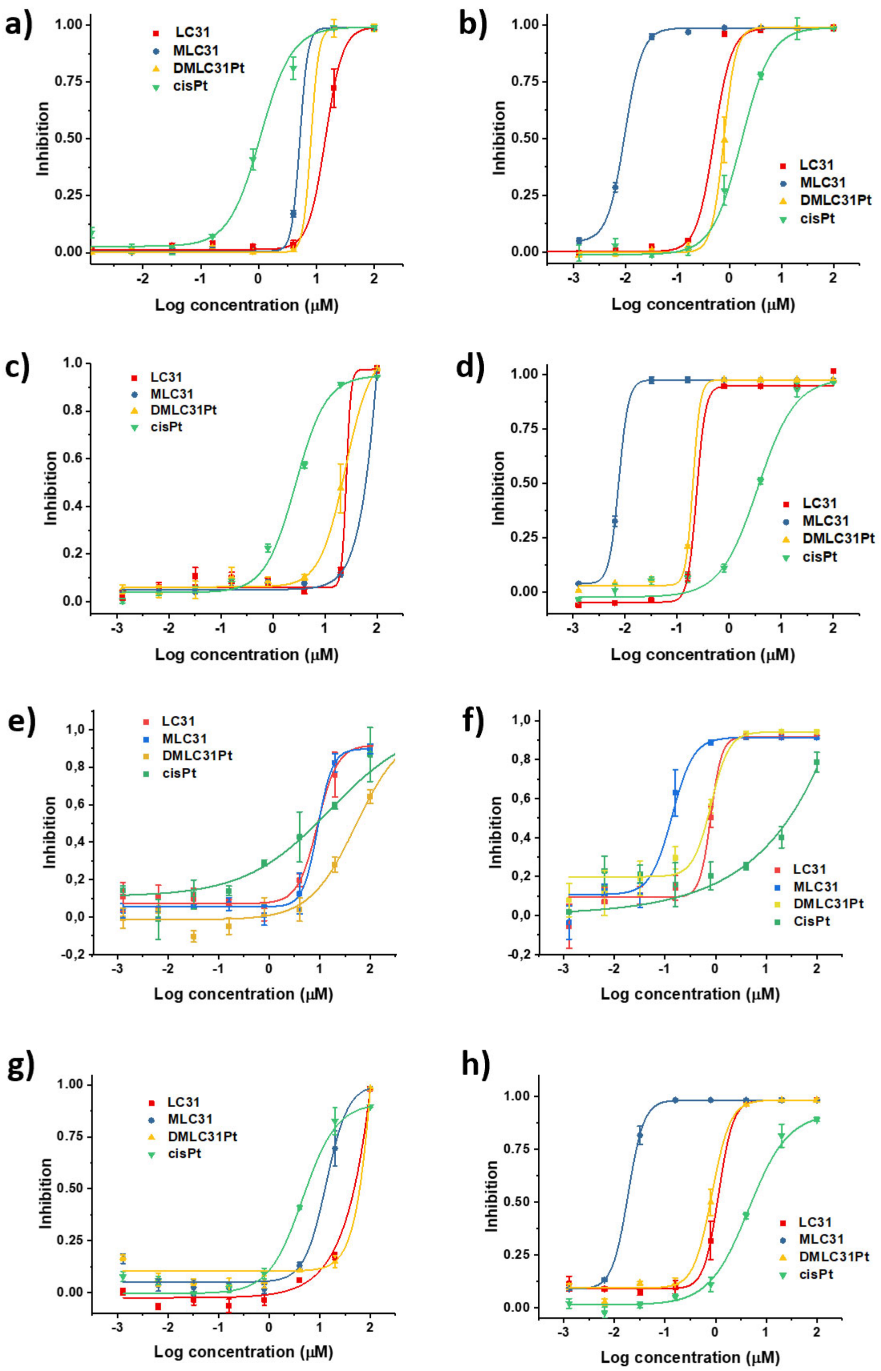

Figure S7. PS-induced cytotoxicity determined by resazurin assays. a) HeLa, c) A2780, e) A2780/CP70, and g) MRC-5 cells were treated with increasing amounts of different PSs for 4 h, then the medium was replaced, and they were further incubated for 72 h. And b) HeLa, d) A2780, f) A2780/CP70, and h) MRC-5 cells were treated with PSs for 4 h, irradiated with NIR light (7 J cm^{-2}) after replacement of the cell medium, and the cell viabilities were determined after 72 h of further incubation. Plotted values are the average of triplicates from three independent experiments with indication of standard error of the mean (SEM).

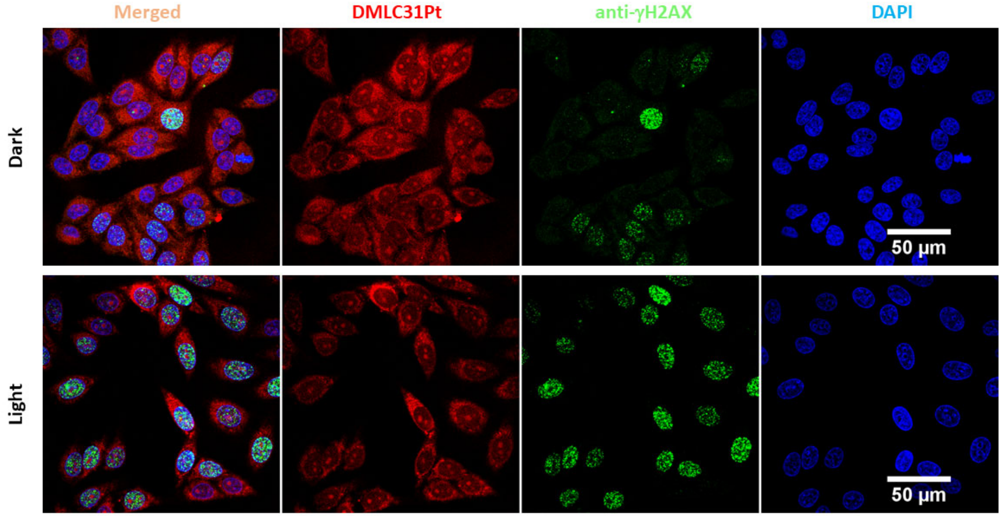

Figure S8. HeLa cells were treated with 500 nM **DMLC31Pt** for 4 h and collected 16 h after being irradiated with NIR light (2.0 mW cm^{-2}, 20 min; 3.6 J cm^{-2}). γH2AX-positive cells (indicative of the presence of DNA double strand breaks) in the dark as well as upon irradiation with light are shown. b) Flow cytometry analysis of the cell cycle affected by co-incubation with PSs. HeLa cells were treated with concentrations of 100 nM of **MLC31**, 200 nM of **DMLC31Pt**, and 500 nM of **cisplatin** for 4 h and collected 16 h and 24 h after being irradiated with NIR light (2.0 mW cm^{-2}, 20 min; 3.6 J cm^{-2}).

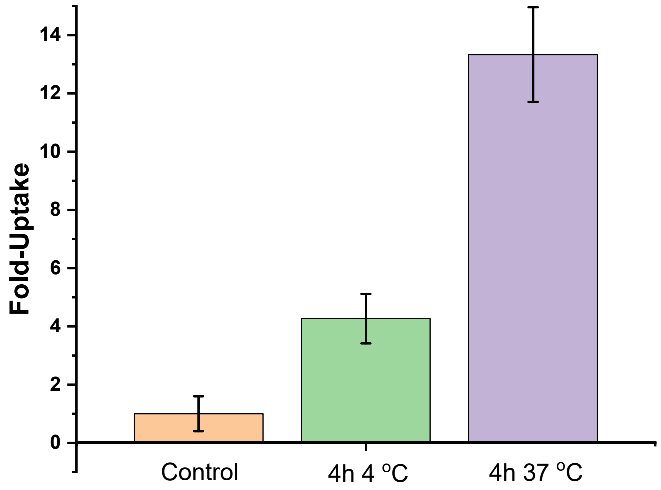

Figure S9. Effects of temperature on the cellular uptake of **MLC31.** HeLa cells were incubated with **MLC31** (2 μM) for 4 h at 4 °C and 37 °C, and the amount of compound taken up was determined by flow cytometry (λ_{ex} = 638 nm, λ_{em} = RL2-A: Alexa Fluor 700).

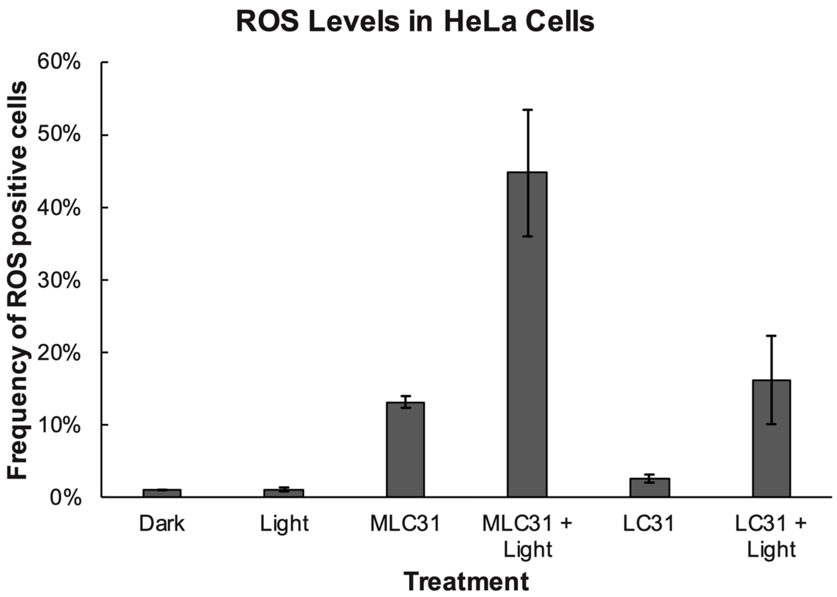

Figure S10. Intracellular ROS generation of **MLC31**, and **LC31** upon irradiation with light. HeLa cells were incubated with either **MLC31** or **LC31** (1 µM) for 4 h at 37 °C. The cells were then irradiated for 20 min with red light, as described in the photocytotoxicity assay procedure. After irradiation, H_2DCF-DA was added to the samples to a final concentration of 50 µM and incubated for 30 min at 37 °C. The samples were analysed on an Attune NxT Cytometer (*Thermo Fisher Scientific*) using the BL1 channel (λ_{ex} = 488 nm, λ_{em} = 515 nm) to measure the DCF fluorescence.

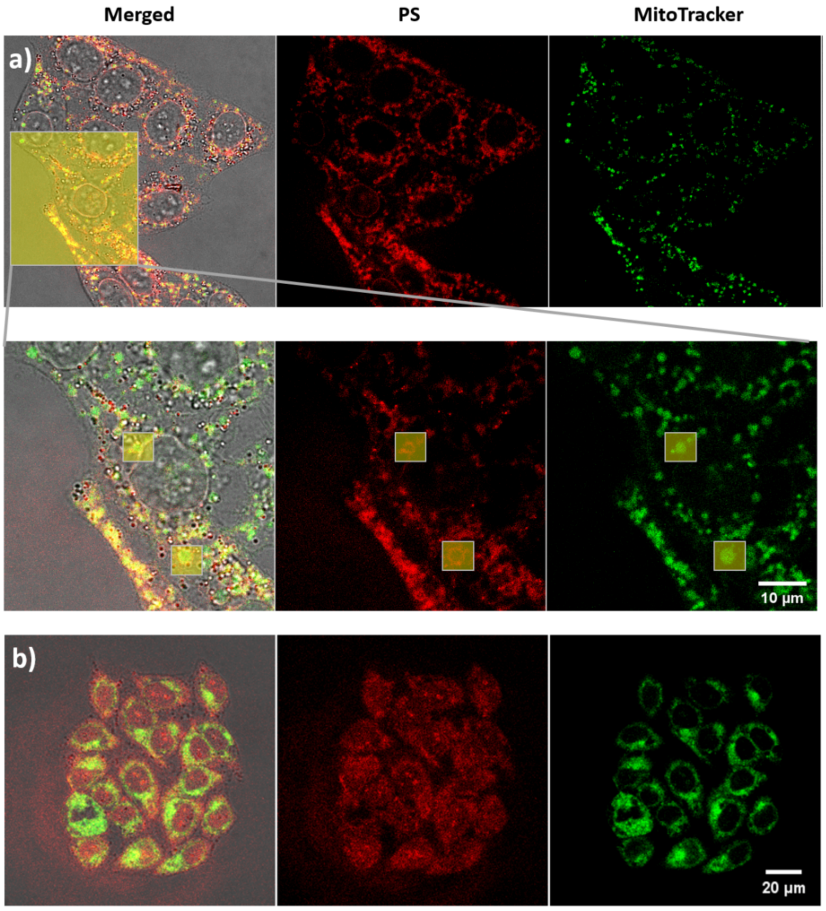

Figure S11. a) Co-localization of **MLC31** (200 nM, 4 h incubation) and MitoTracker Green obtained from live-cell imaging, and the enlarged interested region. b) Co-localization of **DMLC31Pt** (1 μM, 4 h incubation) and MitoTracker Green.

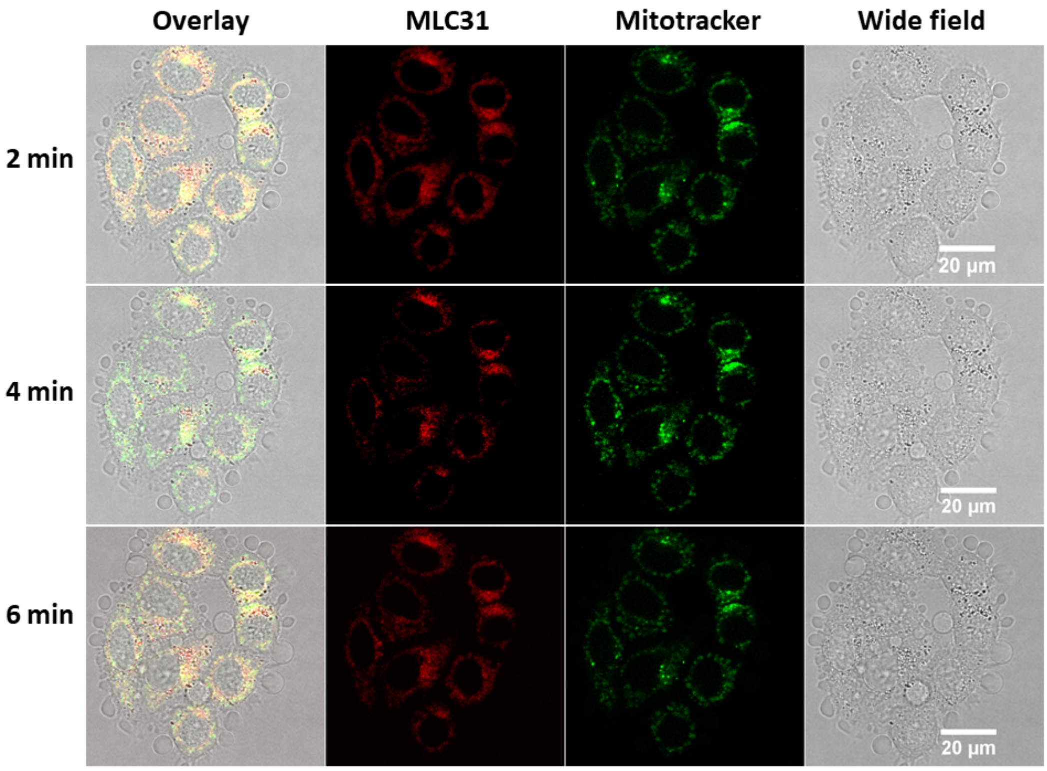

Figure S12. Morphology characterization of apoptosis using live-cell imaging of **MLC31** (200 nM) in HeLa cells with MitoTracker Green staining. Cells were incubated with **MLC31** for 4 h and images were taken after 2, 4, and 6 mins. The left column shows an overlay of red channel (**MLC31**), green channel (MitoTracker Green) and wide field images.

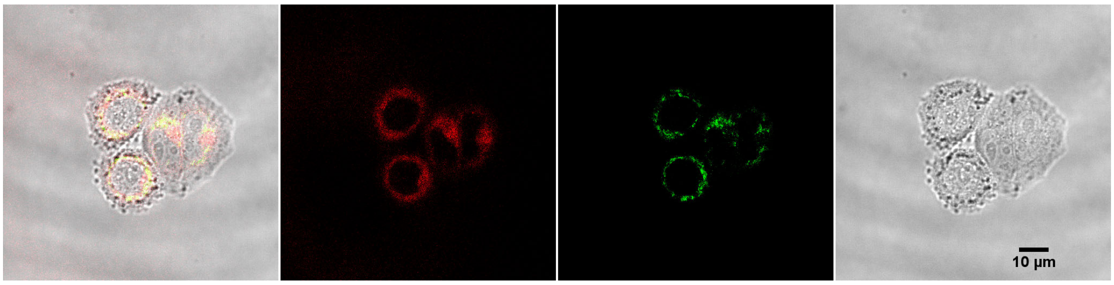

Figure S13. Mitochondria membrane potential change measured by the MMP assay after 20 minutes of 540 and 670 nm light exposure by the Leica SP8 microscope. Snapshots from a time-scale video are shown. From left to right: merged image, **MLC31**, MMP dye and wide field.

References

1. Rigaku Oxford Diffraction *CrysAlis^{Pro} Software system*, 1.171.41; Rigaku Corporation: 2021.
2. Sheldrick, G.M. *SHELXT* - Integrated space-group and crystal-structure determination. *Acta Cryst.* **2015**, *A71*, 3-8, doi:10.1107/S2053273314026370.
3. Sheldrick, G.M. Crystal structure refinement with *SHELXL*. *Acta Cryst.* **2015**, *C71*, 3-8, doi:Doi 10.1107/S2053229614024218.
4. Dolomanov, O.V.; Bourhis, L.J.; Gildea, R.J.; Howard, J.A.K.; Puschmann, H. *OLEX2*: a complete structure solution, refinement and analysis program. *J. Appl. Cryst.* **2009**, *42*, 339-341, doi:10.1107/S0021889808042726.
5. Macrae, C.F.; Sovago, L.; Cottrell, S.J.; Galek, P.T.A.; McCabe, P.; Pidcock, E.; Platings, M.; Shields, G.P.; Stevens, J.S.; Towler, M.; et al. *Mercury 4.0*: from visualization to analysis, design and prediction. *J. Appl. Cryst.* **2020**, *53*, 226-235, doi:10.1107/S1600576719014092.
6. Liu, J.-Y.; Jiang, X.-J.; Fong, W.-P.; Ng, D.K.P. Highly photocytotoxic 1,4-dipegylated zinc(II) phthalocyanines. Effects of the chain length on the *in vitro* photodynamic activities. *Org. Biomol. Chem.* **2008**, *6*, 4560-4566, doi:10.1039/b814627f.
7. Li, F.; Liu, Q.; Liang, Z.; Wang, J.; Pang, M.; Huang, W.; Wu, W.; Hong, Z. Synthesis and biological evaluation of peptide-conjugated phthalocyanine photosensitizers with highly hydrophilic modifications. *Org. Biomol. Chem.* **2016**, *14*, 3409-3422, doi:10.1039/C6OB00122J.
8. Kobayashi, N.; Higashi, R.; Ishii, K.; Hatsusaka, K.; Ohta, K. Aggregation, Complexation with Guest Molecules, and Mesomorphism of Amphiphilic Phthalocyanines Having Four- or Eight Tri(ethylene oxide) Chains. *Bull. Chem. Soc. Jpn.* **1999**, *72*, 1263-1271, doi:10.1246/bcsj.72.1263.
9. Chidawanyika, W.; Nyokong, T. The synthesis and photophysicochemical properties of low-symmetry zinc phthalocyanine analogues. *J. Photochem. Photobiol. A* **2009**, *206*, 169-176, doi:10.1016/j.jphotochem.2009.06.005.
10. Tuncel, S.; Trivella, A.; Atilla, D.; Bennis, K.; Savoie, H.; Albrieux, F.; Delort, L.; Billard, H.; Dubois, V.; Ahsen, V.; et al. Assessing the Dual Activity of a Chalcone-Phthalocyanine Conjugate: Design, Synthesis, and Antivascular and Photodynamic Properties. *Mol. Pharmaceutics* **2013**, *10*, 3706-3716, doi:10.1021/mp400207v.
11. Caron, G.; Ermondi, G.; Gariboldi, M.B.; Monti, E.; Gabano, E.; Ravera, M.; Osella, D. The Relevance of Polar Surface Area (PSA) in Rationalizing Biological Properties of Several *cis*-Diamminemalonatoplatinum(II) Derivatives. *ChemMedChem* **2009**, *4*, 1677-1685, doi:10.1002/cmdc.200900224.
12. Adarsh, N.; Avirah, R.R.; Ramaiah, D. Tuning Photosensitized Singlet Oxygen Generation Efficiency of Novel Aza-BODIPY Dyes. *Org. Lett.* **2010**, *12*, 5720-5723, doi:10.1021/ol102562k.
13. Liu, H.-Y.; Zhao, M.; Qiao, Q.-L.; Lang, H.-J.; Xu, J.-Z.; Xu, Z.-C. Fluorescein-derived fluorescent probe for cellular hydrogen sulfide imaging. *Chin. Chem. Lett.* **2014**, *25*, 1060-1064, doi:10.1016/j.cclet.2014.05.010.
14. Price, M.; Reiners, J.J.; Santiago, A.M.; Kessel, D. Monitoring Singlet Oxygen and Hydroxyl Radical Formation with Fluorescent Probes During Photodynamic Therapy. *Photochem. Photobiol.* **2009**, *85*, 1177-1181, doi:10.1111/j.1751-1097.2009.00555.x.
15. Le, N.A.; Babu, V.; Kalt, M.; Schneider, L.; Schumer, F.; Spingler, B. Photo-stable platinated bacteriochlorins as potent photodynamic agents. *J. Med. Chem.* **2021**, *64*, 6792–6801, doi:10.1021/acs.jmedchem.1c00052.

16. Liu, X.; Xie, J.; Zhang, L.Y.; Chen, H.X.; Gu, Y.; Zhao, J.Q. A novel hypocrellin B derivative designed and synthesized by taking consideration to both drug delivery and biological photodynamic activity. *J. Photochem. Photobiol. B* **2009**, *94*, 171-178, doi:10.1016/j.jphotobiol.2008.11.008.
17. Pucelik, B.; Sułek, A.; Drozd, A.; Stochel, G.; Pereira, M.M.; Pinto, S.M.A.; Arnaut, L.G.; Dąbrowski, J.M. Enhanced Cellular Uptake and Photodynamic Effect with Amphiphilic Fluorinated Porphyrins: The Role of Sulfoester Groups and the Nature of Reactive Oxygen Species. *Int. J. Mol. Sci.* **2020**, *21*, 2786, doi:10.3390/ijms21082786.
18. Schneider, C.A.; Rasband, W.S.; Eliceiri, K.W. NIH Image to ImageJ: 25 years of image analysis. *Nat. Meth.* **2012**, *9*, 671-675, doi:10.1038/nmeth.2089.
